# A Physics Informed Neural Network (PINN) framework for fractional order modeling of Alzheimer's disease

**DOI:** 10.3389/fninf.2026.1748481

**Published:** 2026-02-18

**Authors:** Adnan Mehmood, Muhammad Farman, Farkhanda Afzal, Kottakkaran Sooppy Nisar, Mohammed Altaf Ahmed, Mohamed Hafez

**Affiliations:** 1 CEME, National University of Sciences and Technology (NUST), Islamabad, Pakistan; 2 Department of Mathematics, Mathematics Research Center, Near East University, Northern Cyprus, Türkiye; 3 Research Center of Applied Mathematics, Khazar University, Baku, Azerbaijan; 4 Military College of Signals (MCS), National University of Sciences and Technology (NUST), Islamabad, Pakistan; 5 Department of Mathematics, College of Science and Humanities, Prince Sattam bin Abdulaziz University, Al Kharj, Saudi Arabia; 6 Department of Computer Engineering, College of Computer Engineering & Sciences, Prince Sattam Bin Abdulaziz University, Al Kharj, Saudi Arabia; 7 Faculty of Engineering and Quantity Surviving, INTI International University Colleges, Nilai, Malaysia; 8 Faculty of Management, Shinawatra, Pathum Thani, Thailand

**Keywords:** Alzheimer's disease, health system, machine learning, optimal control, Physics Informed Neural Networks

## Abstract

This study presents a novel fractional order model of Alzheimer's disease (mental disorder) using the Caputo derivative to accurately capture long term memory and hereditary effects in neurodegeneration. The mathematical model incorporates key pathological constituents including neurons, amyloid beta (*A*_β_), tau proteins and microglial responses, allowing detailed simulation of their dynamic interactions. Fundamental properties of the model, including positivity, boundedness, invariant regions and equilibrium points, are rigorously analyzed to ensure biological feasibility. Sensitivity analysis identifies amyloid toxicity as the most influential driver of neuronal loss underscoring its central role in AD progression. Furthermore, a Physics Informed Neural Network (PINN) is developed to approximate system dynamics from noisy observations while ensuring compliance with biological and physical constraints. Compared to standard neural networks the PINN exhibits superior accuracy and robustness especially under data scarcity. By integrating fractional calculus, optimal control and machine learning, this work advances computational modeling of Alzheimer's disease and offers insights into therapeutic optimization.

## 1 Introduction

Fractional order modeling extends traditional integer-order differential equations by incorporating memory and nonlocal dynamics via Caputo or Riemann-Liouville derivatives. Such models integrate the entire history of a process, not just its current state, enabling accurate depiction of delayed responses, long-term persistence and power-law anomalous behaviors. In epidemiology, this approach has enhanced compartmental models like SIR and SEIR, notably in measles transmission, where Caputo-based formulations improve alignment with known incubation periods and long-tail dynamics (Angstmann et al., 2016, 2015; Akuka et al., 2024). Similarly, fractional SEIRD models for COVID-19 incorporate vaccination, quarantine (Bilgil et al., 2022) and reinfection with memory effects, yielding stronger predictive power and dynamic control insights studeid in Hussain et al. (2025).

In cancer modeling, tumor-immune system dynamics under therapeutic interventions have been enriched using fractal fractional derivatives, capturing immune memory, boundedness and stability in ways inaccessible to integer-order frameworks (Nisar et al., 2024a). Parallel advances in fractional pharmacokinetics highlight how drugs exhibiting irregular accumulation and non-exponential decay require fractional kinetics to model their distribution and clearance effectively, informing dosing strategies and toxicity avoidance (Sopasakis et al., 2019). Viscoelastic behavior in biological tissues–especially in immune cells like macrophages–also benefits from fractional modeling: the fractional Kelvin-Voigt model more accurately mirrors viscoelastic response and drug-induced cytoskeletal changes than integer counterparts, offering enhanced diagnostic and therapeutic characterization (Vo and Ekpenyong, 2022). Together, these applications motivate fractional approaches for chronic, history-dependent diseases such as Alzheimer's, where multi-scale interactions among amyloid aggregation, tau pathology, microglial activation and neuronal degradation unfold over long time horizons. A recent fractal fractional Caputo model for AD dynamics rigorously establishes existence, uniqueness and Ulam-Hyers stability and uses fractional Adams-Bashforth schemes that outperform integer-order simulations in reproducing memory-driven trajectories (Yadav et al., 2024). Some more applications and time fractional effect in sense of memory discuses for system (Zhou and Zhang, 2020) and aortic aneurysm (AAA) phenomena studied (Sumelka et al., 2020). Despite their theoretical appeal, numerical and data-driven modeling of fractional systems remains challenging due to the nonlocality of fractional operators, which limits classical solvers and complicates efficient parameter inference. Traditional discretization methods (e.g., finite differences and spectral methods) become expensive and memory-intensive when capturing nonlocal history terms over long time intervals. To address these limitations, Physics Informed Neural Networks (PINNs) embed known differential equations into neural network training by enforcing governing equations as residuals in the loss function, enabling accurate solutions even with scarce or noisy data while avoiding expensive mesh generation and grid-based methods (Raissi et al., 2019). Recent surveys highlight substantial methodological progress in PINNs such as hybrid optimization schemes, adaptive sampling techniques and multi-PDE frameworks, establishing them as versatile and effective methods for tackling both forward and inverse PDE problems across a wide range of scientific applications (Zhang et al., 2025; Farea et al., 2025a; Li, 2025; Kharazmi and Zayernouri, 2021; Murari et al., 2025; Rodrigues, 2024; Shaier et al., 2021; Farea et al., 2025b). Recent advances in fractional modeling and numerical solution techniques have demonstrated the benefits of fractional calculus for capturing memory and nonlocal effects in complex systems. For example, analytical and numerical methods for nonlinear fractional reaction-diffusion equations, such as those arising in blood flow modeling via Laplace-Residual Power Series methods have been developed to efficiently approximate solutions that would be challenging for classical approaches (Ali et al., 2025b). Fractional differential equations have also been applied to brain metabolite dynamics in circadian rhythm models where Caputo-Fabrizio derivatives and series solution techniques provide existence, uniqueness and convergence results beyond integer-order formulations (Ziada and Botros, 2025). In addition, fractional calculus has been used to model nonlinear, multi-dimensional DNA systems, highlighting how fractional models can effectively capture long-range interactions and memory effects that are absent in integer order descriptions (Ali et al., 2025a). The theoretical and numerical developments in these works support the use of fractional models and motivate the incorporation of classical fractional solvers as benchmarks in data-driven methods such as PINNs.

PINNs have been effectively applied to epidemic models such as SIR and SIRD, accurately inferring both state trajectories and time varying transmission rates from noisy outbreak data (Millevoi et al., 2023). Fractional extensions of PINNs further advance this framework by embedding fractional derivatives directly into the loss function, enabling representation of memory effects inherent in fractional PDEs. For example, PINN formulations have been used to solve time fractional Black-Scholes and related fractional diffusion equations by integrating non-integer operators into network residuals (Nuugulu et al., 2025). Other recent work explores enhanced PINN variants for β-conformable fractional differential equations, showing that specialized architecture variants like NRPINN can improve solution quality without domain discretization (Bulut and Yigider, 2025). Moreover, expanded PINN capabilities across biological and epidemiological dynamical systems illustrate the versatility of physics informed approaches when applied to ODEs and coupled systems characterized by known governing laws (Farea et al., 2025b). Nevertheless, critical limitations remain in existing PINN and fractional PINN approaches:

Automatic differentiation cannot directly compute nonlocal fractional operators, which will requires numerical discretization, auxiliary grids or transform techniques that increase complexity and cost, particularly in time fractional problems (Nuugulu et al., 2025; Bulut and Yigider, 2025).

Conventional training of PINNs can exhibit uneven optimization among the different loss components (physics residuals, observational data and boundary/initial conditions), which may cause training to stall or converge to suboptimal solutions. While recent studies introduce adaptive loss-weighting and sampling schemes to address this, more comprehensive methodological advances are still an unresolved research problem (Farea et al., 2025a) and some applications related problem is given in Lawal et al. (2022), Sopasakis and Kalliadasis (2019), Srivastava (2024), and Farea et al. (2025c).

Techniques using Monte Carlo sampling or structured sampling modules can reduce grid dependence, but often at the cost of estimator variance and sensitivity to hyperparameters, which limits robustness in large scale, high dimensional systems (Ahmad et al., 2025).

Many existing fractional PINN studies focus on specialized PDEs or prototype systems, rather than unified, scalable frameworks capable of handling multi scale biological systems with intertwined memory effects and noisy observational data, such as those found in Alzheimer's progression.

These limitations underscore the need for robust and scalable fractional PINN frameworks that can efficiently balance physics and data objectives, improve training stability and enable reliable parameter inference even under data scarcity. To bridge these gaps, foundational and emerging studies collectively underscore the adaptability and strength of PINNs in modeling complex dynamical systems, ranging from epidemic scenarios to tumor progression and systems biology. PINNs unite mechanistic knowledge and data driven learning with fractional, time varying and multi phase dynamics making them an ideal tool for realistic disease modeling across domains.

The structure of this paper is organized as follows. In Section 2, we introduce the mathematical preliminaries of fractional calculus and present the physics informed neural network (PINN) framework used to approximate fractional-order dynamical systems. Section 3 formulates the proposed fractional-order Alzheimer's disease model and provides a detailed theoretical analysis, including positivity, boundedness, existence, uniqueness, equilibrium points and stability properties. Section 4 presents sensitivity analysis and numerical simulations that illustrate the influence of key biological parameters and fractional order on disease progression. In Section 5, a fractional PINN-based optimal control framework is developed and its performance is analyzed. In addition, the proposed fractional PINN is benchmarked against a classical Grünwald Letnikov numerical solver to verify its ability to reproduce controlled fractional dynamics. Finally, the concluding section summarizes the findings and gives potential directions for future research.

## 2 Mathematical framework: Physics Informed Neural Networks

We consider a system of nonlinear ordinary or fractional differential equations governing the evolution of biological or physical quantities. Let D=[0,T] denote the temporal domain of interest and let *u*(*t*) ∈ ℝ^*n*^ represent the vector of state variables (e.g., neuronal population, amyloid-β concentration, etc.). The general form of the governing system is given by:


$$\begin{array}{l} F\left(t,u\left(t\right){,}^{C}D_{t}^{\sigma }u\left(t\right);\theta \right)=0,\;t\in D, \end{array}$$ (1)

with initial condition:


$$\begin{array}{l} u\left(0\right)=u_{0}\in {\mathbb{R}}^{n}. \end{array}$$ (2)

Here:

- F is a (possibly nonlinear) differential operator parameterized by θ,
- DtσCu(t) denotes the Caputo fractional derivative of order σ ∈ (0, 1],
- θ is a set of known or learnable parameters.

Definition 2.1. A Physics Informed Neural Network (PINN) is a neural network $u_{\phi }:Darrow{\mathbb{R}}^{n}$ with parameters ϕ, trained to approximate the true solution *u*(*t*) such that:

1. It minimizes the discrepancy with available data,
2. It satisfies the governing differential equation(s).

- $T_{d}={\left\{t_{i}^{\left(d\right)}\right\}}_{i=1}^{N_{d}}\subset D$ be the set of data points,
- $T_{c}={\left\{t_{j}^{\left(c\right)}\right\}}_{j=1}^{N_{c}}\subset D$ be the set of collocation points.

The total loss function used to train *u*_ϕ_ is:


$$\begin{array}{l} L\left(\phi \right)=L_{\text{data}}\left(\phi \right)+\lambda_{\text{phys}}L_{\text{phys}}\left(\phi \right), \end{array}$$ (3)

where:


$$\begin{array}{l} L_{\text{data}}\left(\phi \right)=\frac{1}{N_{d}}\overset{N_{d}}{\underset{i=1}{\sum }}\|u_{\phi }\left(t_{i}^{\left(d\right)}\right)-u^{\text{obs}}\left(t_{i}^{\left(d\right)}\right){\|}^{2}, \end{array}$$ (4)

$$\begin{array}{l} L_{\text{phys}}\left(\phi \right)=\frac{1}{N_{c}}\overset{N_{c}}{\underset{j=1}{\sum }}\|F\left(t_{j}^{\left(c\right)},u_{\phi }\left(t_{j}^{\left(c\right)}\right){,}^{C}D_{t}^{\sigma }u_{\phi }\left(t_{j}^{\left(c\right)}\right);\theta \right){\|}^{2}, \end{array}$$ (5)

and λ_phys_ > 0 is a regularization parameter.

Remark 1. In practice, the Caputo derivative is approximated numerically using a discrete convolution formula such as the Grünwald-Letnikov scheme:


DtσCu(tn)≈1Δtσ∑k=0nwk(σ)u(tn−k), (6)

where:

- Δ*t* is the time step size,
- $w_{k}^{\left(\sigma \right)}={\left(-1\right)}^{k}\left(\begin{array}{c} \sigma \\ k \end{array}\right)$ are fractional weights,
- $\left(\begin{array}{c} \sigma \\ k \end{array}\right)=\frac{\Gamma (\sigma +1)}{\Gamma (k+1)\Gamma (\sigma -k+1)}$.

This allows the PINN to model nonlocal memory effects present in biological systems.

Definition 2.2. The residual function evaluated at the collocation point $t_{j}^{\left(c\right)}$ is given by:


$$\begin{array}{l} R_{\phi }\left(t_{j}^{\left(c\right)}\right):=F\left(t_{j}^{\left(c\right)},u_{\phi }\left(t_{j}^{\left(c\right)}\right){,}^{C}D_{t}^{\sigma }u_{\phi }\left(t_{j}^{\left(c\right)}\right);\theta \right). \end{array}$$ (7)

The physics loss term is then defined as:


$$\begin{array}{l} L_{\text{phys}}\left(\phi \right)=\frac{1}{N_{c}}\overset{N_{c}}{\underset{j=1}{\sum }}\|R_{\phi }\left(t_{j}^{\left(c\right)}\right){\|}^{2}. \end{array}$$ (8)

Remark 2. Under the activation functions (e.g., tanh, ReLU), neural networks are universal function approximators. Thus, given sufficient, *u*_ϕ_(*t*) can approximate the true solution *u*(*t*) arbitrarily well, provided that the optimization landscape is well conditioned.

Definition 2.3. (Kilbas et al., 2006) Assume that [*a, b*] ⊂ ℝ. Then the fractional integral of order σ for *g* ∈ *L*^1^([*a, b*], ℝ) can be written as:


$$I_{t}^{\sigma }g\left(t\right)=\frac{1}{\Gamma \left(\sigma \right)}\int_{0}^{t}{\left(t-v\right)}^{\left(1-\sigma \right)}g\left(v\right)dv$$

where *t* > 0, σ > 0 and integral on the right side is point-wise defined on ℝ^+^, ℝ^+^ = [0, ∞).

Definition 2.4. (Podlubny, 1999) Let *g* be a continuous function on [0, *T*]. The derivative of Caputo can be written as


Dtσ0Cg(t)=1Γ(n−σ)[∫0t(t−v)n−σ−1dndvng(t)(v)dv]

where *n* = ⌊σ⌋+1 and ⌊σ⌋ be the integer of β. Where 0 < σ < 1 then the Caputo derivatives will be:


Dtσ0Cg(t)=1Γ(n−σ)[∫0t(t−v)−σg′(t)(v)dv]

Lemma 1. (Bansal et al., 2023) Let *t*≥*t*_0_ and let $F:\left[t_{0},\infty \right)\to {\mathbb{R}}^{+}$ be a continuous function. For any σ ∈ (0, 1) and any constant F^*^ ∈ ℝ^+^, the following inequality holds:


DtσC​(F(t)−F*−F*ln​F(t)F*) ≤ (1−F*F(t))DtσCF(t),

where DtσC denotes the Caputo fractional derivative of order σ.

## 3 Fractional order model

Amyloid and tau are the two primary proteins that are hypothesized to obstruct brain cell to cell communication. The amyloid beta peptide accumulates in Alzheimer's disease and deposits as plaques around brain vasculature and neuronal cells. This deposition is linked to a decrease in neural function, which impairs memory and cognition and affects daily functioning including speaking, writing and thinking. Once amyloid beta has accumulated to a certain degree, aberrant tau begins to surge, following the initial appearance of amyloid beta clusters. Then, a positive feedback loop takes place leading to an increase in aberrant tau and amyloid beta formation. Numerous bacteria and viruses, as well as tau tangles and amyloid beta plaques can cause neuro-inflammation. The resident innate immune cells of the central nervous system known as microglia, have the ability to trigger the activation of inflammatory pathways and change their physiological function, which can accelerate the progression of disease. Microglia are believed to become activated when harmful amyloid beta and tau proteins are present. Toxic proteins and other detritus are removed from dead and dying cells by microglia. Neuronal dysfunction, damage and loss may arise from chronic inflammation caused by microglia's inability to keep up with everything that needs to be cleaned.

The model is given by:


D0σCFN(t)=ΠN+ρTμ−αFNAβ−ϕ1FND0σCIN(t)=αFNAβ−β1INMρ−(γ+ϕ2)IND0σCAβ(t)=γIN−β2AβMρ−(dβ+κ)AβD0σCTμ(t)=κAβ−β3TμMρ−(dμ+ρ)TμD0σCMρ(t)=(β1IN+β2Aβ+β3Tμ)Mρ−ϕ3Mρ (9)

The model parameters in Table 1 represents the biologically meaningful processes governing neuronal survival, protein aggregation and immune response. Parameters such as α, κ and γ quantify the interaction strength between amyloid beta, tau protein and neuronal compartments, while β_1_, β_2_ and β_3_ describe microglial clearance rates of infected neurons and toxic proteins. Some parameters are assumed or estimated due to their limited availability of precise experimental measurements, however, their values are chosen within biologically plausible ranges consistent with existing literature. The small initial values assigned to amyloid beta and tau protein concentrations reflects an early stage pathological conditions and ensure physiological realism of the simulated trajectories.

**Table 1:** Initial variables states and parameters values.

**Parameter**	**Description**	**Value**	**Unit**	**Source**
*F*_*N*_	Functional brain neurons	0.14	g/ml	Hao and Friedman, 2016
*I*_*N*_	Infected brain neurons	0	g/ml	Hao and Friedman, 2016
*A*_β_	Amyloid beta concentration in brain	0.000001	g/ml	Hao and Friedman, 2016
*T*_μ_	Tau protein concentration in brain	0.000001	g/ml	Estimated
*M*_ρ_	Microglia concentration in brain	0.02	g/ml	Hao and Friedman, 2016
Π_*N*_	Rate of neuron production in brain	1	-	Assumed
ρ	Rate of Neuro degeneration from Tau protein	0.025	Per day	Petrella et al., 2019
α	Rate of Amyloid beta cascade growth in neurons	0.08	Per day	Petrella et al., 2019
ϕ_1_	Natural death rate of neurons in brain	0.02	per year	Assumed
β_1_	Killing rate of infected neurons by Microglia	0.06	Per day	Hao and Friedman, 2016
γ	Clearance of neurons by Amyloid beta	0.00017	Per day	Hao and Friedman, 2016
ϕ_2_	Death rate of infected neurons	0.00019	Per day	Hao and Friedman, 2016
β_2_	Clearance rate of Amyloid beta by Microglia	0.002	Per day	Hao and Friedman, 2016
*d*_β_	Proteolytic degradation rate of Amyloid beta	9.51	Per day	Hao and Friedman, 2016
κ	Initiating rate of Tau protein by Amyloid beta	0.025	Per day	Petrella et al., 2019
β_3_	Clearance rate of Tau protein by Microglia	0.001	Per day	Estimated
*d*_μ_	Natural degradation rate of tau protein	0.277	Per day	Hao and Friedman, 2016
ϕ_3_	Death rate of Microglia	0.015	Per day	Hao and Friedman, 2016

### 3.1 Positivity and boundedness of solutions

For numerous forms of differential operators, including non-integer and integer orders, we provide a comprehensive analysis that substantiates the requirements for maintaining the positiveness of the suggested model solutions. To accomplish this, Now, define the Norm as,


$$\begin{array}{l} \left\|B{\|}_{\infty }=\underset{t\in D_{B}}{\sup}|B\left(t\right)\right| \end{array}$$ (10)

*D*_*B*_ represents the domain of *B*. The definition given above can be used to generate the following inequality for the function *F*_*N*_:


$$\begin{array}{l} F_{N}\left(t\right)=\Pi_{N}+\rho T_{\mu }-\alpha F_{N}A_{\beta }-\phi_{1}F_{N},\;\forall t\geq 0\\ \;\geq -\left(\alpha A_{\beta }+\phi_{1}\right)F_{N},\;\forall t\geq 0\\ \;\geq -\left(\alpha \underset{t\in D_{O}}{\sup}|A_{\beta }|+\phi_{1}\right)F_{N},\;\forall t\geq 0\\ \;\geq -\left(\alpha \|A_{\beta }{\|}_{\infty }+\phi_{1}\right)F_{N},\;\forall t\geq 0 \end{array}$$ (11)

This yields


$$\begin{array}{l} F_{N}\left(t\right)\geq F_{N_{0}}\exp^{-\left(\alpha \|A_{\beta }{\|}_{\infty }+\phi_{1}\right)t},\;\forall t\geq 0 \end{array}$$ (12)

For the other functions:


$$\begin{array}{l} I_{N}\left(t\right)=\alpha F_{N}A_{\beta }-\beta_{1}I_{N}M_{\rho }-\left(\gamma +\phi_{2}\right)I_{N},\;\forall t\geq 0\\ \;\geq -\left(\beta_{1}M_{\rho }+\gamma +\phi_{2}\right)I_{N},\;\forall t\geq 0\\ \;\geq -\left(\beta_{1}\|M_{\rho }{\|}_{\infty }+\phi_{2}\right)I_{N},\;\forall t\geq 0 \end{array}$$ (13)

This yields


$$\begin{array}{l} I_{N}\left(t\right)\geq I_{N_{0}}\exp^{-\left(\beta \|M_{\rho }{\|}_{\infty }+\phi_{2}\right)t},\;\forall t\geq 0. \end{array}$$ (14)

### 3.2 Positive solutions with non local operators

Here, we show that solutions with non-local operators are positive for a fractional calculus model. If every initial condition is met for non-local operators, then every solution is positive. The Caputo derivative gives,


$$\begin{array}{l} F_{N}(t)\geq F_{N}(0)E_{\sigma }\left(-\left(\alpha {\left\|A_{\beta }\right\|}_{\infty }+\phi_{1}\right)t^{\sigma }\right),\;\forall t\geq 0\\ I_{N}(t)\geq I_{N}(0)E_{\sigma }\left(-\left(\beta {\left\|M_{\rho }\right\|}_{\infty }+\phi_{2}\right)t^{\sigma }\right),\;\forall t\geq 0\\ A_{\beta }(t)\geq A_{\beta }(0)E_{\sigma }\left(-\left(\beta_{2}{\left\|M_{\rho }\right\|}_{\infty }+d_{\beta }+\kappa \right)t^{\sigma }\right),\;\forall t\geq 0\\ T_{\mu }(t)\geq T_{\mu }(0)E_{\sigma }\left(-\left(\beta_{3}{\left\|M_{\rho }\right\|}_{\infty }+d_{\mu }+\rho \right)t^{\sigma }\right),\;\forall t\geq 0\\ M_{\rho }(t)\geq M_{\rho }(0)E_{\sigma }((\beta_{1}{\left\|I_{N}\right\|}_{\infty }+\beta_{2}{\left\|A_{\beta }\right\|}_{\infty }+\beta_{3}{\left\|T_{\mu }\right\|}_{\infty }\\ \;-\phi_{3})t^{\sigma }),\;\forall t\geq 0. \end{array}$$ (15)

### 3.3 Existence and uniqueness analysis

Now we use fixed point theory to investigate the existence and uniqueness of our model. Banach's contraction theorem will guaranties the model's singularity, whereas Schauder's fixed point theorem will guaranties its existence. Theorems will show that the our model has a unique solution are important since they suggest that your problem can be solved in a unique way. We can estimate a solution using numerical methods once we are certain that it exists. By using a fractional derivative for 0 < η ≤ 1 in the Caputo sense, system (9) can be made more generic. Let


$$\begin{array}{l} \rho_{1}\left(t,F_{N}\right)=\Pi_{N}+\rho T_{\mu }-\alpha F_{N}A_{\beta }-\phi_{1}F_{N}\\ \rho_{2}\left(t,I_{N}\right)=\alpha F_{N}A_{\beta }-\beta_{1}I_{N}M_{\rho }-\left(\gamma +\phi_{2}\right)I_{N}\\ \rho_{3}\left(t,A_{\beta }\right)=\gamma I_{N}-\beta_{2}A_{\beta }M_{\rho }-\left(d_{\beta }+\kappa \right)A_{\beta }\\ \rho_{4}\left(t,T_{\mu }\right)=\kappa A_{\beta }-\beta_{3}T_{\mu }M_{\rho }-\left(d_{\mu }+\rho \right)T_{\mu }\\ \rho_{5}\left(t,M_{\rho }\right)=\left(\beta_{1}I_{N}+\beta_{2}A_{\beta }+\beta_{3}T_{\mu }\right)M_{\rho }-\phi_{3}M_{\rho } \end{array}$$ (16)

With the fractional integral and initial condition, the situation is:


$$\begin{array}{l} F_{N}\left(t\right)=F_{N_{0}}+\frac{1}{\Gamma \left(\sigma \right)}\int_{0}^{t}{\left(t-v\right)}^{\sigma -1}\rho_{1}\left(t,F_{N}\right)dv\\ I_{N}\left(t\right)=I_{N_{0}}+\frac{1}{\Gamma \left(\sigma \right)}\int_{0}^{t}{\left(t-v\right)}^{\sigma -1}\rho_{2}\left(t,I_{N}\right)dv\\ A_{\beta }\left(t\right)=A_{\beta_{0}}+\frac{1}{\Gamma \left(\sigma \right)}\int_{0}^{t}{\left(t-v\right)}^{\sigma -1}\rho_{3}\left(t,A_{\beta }\right)dv\\ T_{\mu }\left(t\right)=T_{\mu_{0}}+\frac{1}{\Gamma \left(\sigma \right)}\int_{0}^{t}{\left(t-v\right)}^{\sigma -1}\rho_{3}\left(t,T_{\mu }\right)dv\\ M_{\rho }\left(t\right)=M_{\rho_{0}}+\frac{1}{\Gamma \left(\sigma \right)}\int_{0}^{t}{\left(t-v\right)}^{\sigma -1}\rho_{4}\left(t,M_{\rho }\right)dv \end{array}$$ (17)

Let


$$\Theta (t)=\{\begin{array}{l} F_{N}(t)\\ I_{N}(t)\\ A_{\beta }(t)\\ T_{\mu }(t)\\ M_{\rho }(t) \end{array},\Theta_{0}=\{\begin{array}{l} F_{N_{0}}\\ I_{N_{0}}\\ A_{\beta_{0}}\\ T_{\mu_{0}}\\ M_{\rho_{0}} \end{array},ℵ(\xi ,\Theta (\xi ))=\{\begin{array}{l} \rho_{1}\left(t,F_{N}\right)\\ \rho_{2}\left(t,I_{N}\right)\\ \rho_{3}\left(t,A_{\beta }\right)\\ \rho_{4}\left(t,T_{\mu }\right)\\ \rho_{5}\left(t,M_{\rho }\right) \end{array}$$ (18)

Thus,


$$\begin{array}{l} \Theta \left(t\right)=\Theta_{0}+\frac{1}{\Gamma \left(\sigma \right)}\int_{0}^{t}{\left(t-v\right)}^{\sigma -1}ℵ\left(v,\Theta \left(v\right)\right)dv \end{array}$$ (19)

Now take a Banach space with a norm ℘[0, ℝ] = χ:


$$\begin{array}{l} \|F_{N},I_{N},A_{\beta },T_{\mu },M_{\rho }\|=\underset{t\in \left[0,\mathbb{R}\right]}{\max}\left[\left|F_{N},I_{N},A_{\beta },T_{\mu },M_{\rho }\right|\right] \end{array}$$ (20)

Let a mapping defined as ◇: *χ* → *χ*


$$\begin{array}{l} ◇\Theta \left(t\right)=\Theta_{0}+\frac{1}{\Gamma \left(\sigma \right)}\int_{0}^{t}{\left(t-v\right)}^{\sigma -1}ℵ\left(v,\Theta \left(v\right)\right)dv \end{array}$$ (21)

Furthermore, we subject a nonlinear function to the next proposition:

- (P1) Constants ψ_*m*_, ψ_*m*_ > 0 exist such that

$$|ℵ(t,\Theta (t)|\leq \psi_{m}|\Theta (t)|+\psi_{m}$$ (22)

- (P2) Every $\Theta ,\bar{\Theta }\in \chi$ has a constant *L*_*m*_ > 0 according to which

$$\begin{array}{l} \left|ℵ\left(t,\bar{\Theta }\right)-ℵ\left(t,{\bar{\Theta }}_{1}\right)|\leq L_{m}|\bar{\Theta }-{\bar{\Theta }}_{1}\right| \end{array}$$ (23)

Theorem 1. If the hypotheses (P1) is correct, then the system (9) has at least one solution.

*Proof*. Let χ = *C*([0, τ], ℝ^*n*^) with the sup-norm


$$\begin{array}{l} \|\Theta \|=\underset{t\in \left[0,\tau \right]}{\sup}\|\Theta \left(t\right)\|. \end{array}$$ (24)

Assume that the nonlinear function ℵ:[0, τ] × ℝ^*n*^ → ℝ^*n*^ satisfies the growth and Lipschitz conditions


$$\|ℵ(t,\Theta )\|\leq \psi_{m}(\|\Theta \|+1),\;\|ℵ(t,\Theta_{1})-ℵ(t,\Theta_{2})\|\leq L\|\Theta_{1}-\Theta_{2}\|,$$ (25)

for all *t* ∈ [0, τ] and Θ, Θ_1_, Θ_2_ ∈ χ, where ψ_*m*_, *L* ≥ 0 are constants.

Define


$$\psi \geq \underset{t\in [0,\tau ]}{\max}\frac{\|\Theta_{0}\|+\frac{\psi_{m}\tau^{\sigma }}{\Gamma (\sigma +1)}}{1-\frac{\psi_{m}\tau^{\sigma }}{\Gamma (\sigma +1)}},$$ (26)

and let


$$\begin{array}{l} B:=\left\{\Theta \in \chi :\|\Theta \|\leq \psi \right\}. \end{array}$$ (27)

Clearly, *B* is closed, convex and bounded in χ.

Now define the operator T:χ→χ by


$$\begin{array}{l} \left(T\Theta \right)\left(t\right)=\Theta_{0}+\frac{1}{\Gamma \left(\sigma \right)}\int_{0}^{t}{\left(t-v\right)}^{\sigma -1}ℵ\left(v,\Theta \left(v\right)\right)dv,\;t\in \left[0,\tau \right]. \end{array}$$ (28)

T **maps** **B** **into** **B**. For Θ ∈ *B* and *t* ∈ [0, τ],


$$\begin{array}{l} \|(T\Theta )(t)\|\leq \|\Theta_{0}\|+\frac{1}{\Gamma (\sigma )}\int_{0}^{t}{\left(t-v\right)}^{\sigma -1}\|ℵ(v,\Theta (v))\|dv\\ \;\leq \|\Theta_{0}\|+\frac{1}{\Gamma (\sigma )}\int_{0}^{t}{\left(t-v\right)}^{\sigma -1}\psi_{m}(\|\Theta \|+1)dv\\ \;\leq \|\Theta_{0}\|+(\psi_{m}\|\Theta \|+\psi_{m})\frac{\tau^{\sigma }}{\Gamma (\sigma +1)}\\ \;\leq \|\Theta_{0}\|+(\psi_{m}\psi +\psi_{m})\frac{\tau^{\sigma }}{\Gamma (\sigma +1)}\leq \psi , \end{array}$$ (29)

by the choice of ψ. Thus, T(B)⊆B.

T **is continuous and relatively compact**. Let *t*_1_<*t*_2_ ∈ [0, τ]. For Θ ∈ *B*,


$$\begin{array}{l} \left\|(T\Theta )(t_{2})-(T\Theta )(t_{1})\right\|\\ \;=\frac{1}{\Gamma (\sigma )}\|\int_{0}^{t_{2}}{\left(t_{2}-v\right)}^{\sigma -1}ℵ(v,\Theta (v))dv\\ \;-\int_{0}^{t_{1}}{\left(t_{1}-v\right)}^{\sigma -1}ℵ(v,\Theta (v))dv\|\\ \leq \frac{1}{\Gamma (\sigma )}\int_{0}^{t_{1}}[(t_{2}-v{)}^{\sigma -1}-(t_{1}-v{)}^{\sigma -1}]\|\\ ℵ(v,\Theta (v))\|dv\\ \;+\frac{1}{\Gamma (\sigma )}\int_{t_{1}}^{t_{2}}{\left(t_{2}-v\right)}^{\sigma -1}\|ℵ(v,\Theta (v))\|dv\\ \;\leq (\psi_{m}\psi +\psi_{m})\frac{t_{2}^{\sigma }-t_{1}^{\sigma }}{\Gamma (\sigma +1)}. \end{array}$$ (30)

As *t*_2_→*t*_1_, the right-hand side tends to 0, so TΘ is equicontinuous on [0, τ]. Since ‖(TΘ)(t)‖≤ψ for all *t*, the set T(B) is uniformly bounded. By the Arzelà-Ascoli theorem T(B) is relatively compact in χ. Continuity of T follows from dominated convergence.

#### 3.3.1 Existence of a solution

The operator T is continuous, maps *B* into a relatively compact subset of *B* and *B* is closed, bounded and convex. Hence, by *Schauder's fixed point theorem* T has at least one fixed point Θ^*^ ∈ *B*. Thus, the system has at least one solution.

#### 3.3.2 Uniqueness under a contraction condition

If the Lipschitz constant *L* satisfies


$$\begin{array}{l} L\frac{\tau^{\sigma }}{\Gamma \left(\sigma +1\right)}<1, \end{array}$$ (31)

then for Θ_1_, Θ_2_ ∈ *B*,


$$\begin{array}{l} \left\|\left(T\Theta_{1}\right)\left(t\right)-\left(T\Theta_{2}\right)\left(t\right)\|\leq \frac{1}{\Gamma \left(\sigma \right)}\int_{0}^{t}{\left(t-v\right)}^{\sigma -1}\right\|\\ \;ℵ\left(v,\Theta_{1}\left(v\right)\right)-ℵ\left(v,\Theta_{2}\left(v\right)\right)\|dv\\ \;\leq L\|\Theta_{1}-\Theta_{2}\|\frac{\tau^{\sigma }}{\Gamma \left(\sigma +1\right)}. \end{array}$$ (32)

Taking the supremum over *t* ∈ [0, τ] gives


$$\begin{array}{l} \|T\Theta_{1}-T\Theta_{2}\|\leq L\frac{\tau^{\sigma }}{\Gamma \left(\sigma +1\right)}\|\Theta_{1}-\Theta_{2}\|. \end{array}$$ (33)

Thus, T is a contraction. By the *Banach fixed point theorem* the fixed point is unique.

Therefore, the system has at least one solution (by Schauder's theorem) and under the contraction condition this solution is unique (by Banach's theorem).

Theorem 2. Assume that the system (9) has a unique solution if the conditions (P2) are satisfied.

*Proof*. Assume that $\bar{\Theta },{\bar{\Theta }}_{1}\in \chi$. Then,


$$\begin{array}{l} \|◇\left(\bar{\Theta }\right)-◇\left({\bar{\Theta }}_{1}\right)\|=\underset{t\in \left[0,\tau \right]}{\max}|\frac{1}{\Gamma \left(\sigma \right)}\int_{0}^{t}{\left(t_{2}-v\right)}^{\sigma -1}ℵ\left(v,\bar{\Theta }\left(v\right)\right)dv.\\ \;.-\frac{1}{\Gamma \left(\sigma \right)}\int_{0}^{t}{\left(t_{1}-v\right)}^{\sigma -1}ℵ\left(v,{\bar{\Theta }}_{1}\left(v\right)\right)dv| \end{array}$$ (34)

$$\begin{array}{l} \;\leq \frac{\tau^{\sigma }}{\Gamma \left(\sigma +1\right)}L_{m}|\bar{\Theta }-{\bar{\Theta }}_{1}|. \end{array}$$ (35)

◇ is consequently a contraction. The system (9) has a unique solution according to the Banach fixed point theorem.

### 3.4 Equilibrium point analysis

In this part, the equilibrium points will be examined. The equilibrium points of the system (9) are: When $I_{N}^{0}=0$, the disease free equilibrium points (DEF) will be,


$$\begin{array}{l} E^{0}=\left(F_{N}^{0},I_{N}^{0},A_{\beta }^{0},T_{\mu }^{0},M_{\rho }^{0}\right) \end{array}$$ (36)

If $I_{N}^{0}=0$, then $F_{N}^{0}=\frac{\Pi_{N}}{\phi_{1}},A_{\beta }^{0}=0$ and $T_{\mu }^{0}=0$, represent the disease free equilibrium points (DEF), then,


$$\begin{array}{l} E^{0}=\left(\frac{\Pi_{N}}{\phi_{1}},0,0,0\right) \end{array}$$ (37)

Now, the endemic equilibrium points are:


$$\begin{array}{l} E^{*}=\left(F_{N}^{*},I_{N}^{*},A_{\beta }^{*},T_{\mu }^{*}\right)\\ F_{N}^{*}=\frac{\gamma I_{N}\kappa \rho +\Pi_{N}d_{\mu }d_{\beta }+\Pi_{N}d_{\beta }+\Pi_{N}d_{\mu }\kappa +\Pi_{N}\rho \kappa }{\alpha d_{\mu }\gamma I_{N}+\alpha \gamma I_{N}\rho +\phi_{1}d_{\mu }d_{\beta }+\phi_{1}d_{\beta }\rho +\phi_{1}d_{\mu }\kappa +\phi_{1}\kappa \rho }\\ \;I_{N}^{*}=I_{N}^{*}\\ \;A_{\beta }=\frac{\gamma I_{N}^{*}}{d_{\beta }+\kappa }\\ \;T_{\mu }^{*}=\frac{\kappa \gamma I_{N}^{*}}{\left(d_{\mu }+\rho \right)\left(d_{\beta }+\kappa \right)} \end{array}$$ (38)

The proposed system is absolutely stable.

### 3.5 The reproduction number

We now compute the basic reproduction number *R*_0_ for the Alzheimer's model using the next-generation matrix method.

The infected states are


$$X\left(t\right)={\left(I_{N}\left(t\right),A_{\beta }\left(t\right),T_{\mu }\left(t\right)\right)}^{⊤}.$$

At the disease-free equilibrium (DFE), we have


$$I_{N}^{*}=A_{\beta }^{*}=T_{\mu }^{*}=0,\;F_{N}^{*}=\frac{\Pi_{N}}{\phi_{1}}.$$

Linearizing the equations around the DFE gives:


$$\begin{array}{l} D_{t}^{\alpha }I_{N}=\alpha_{\text{inf}}F_{N}^{*}A_{\beta }-\left(\gamma_{ab}+\phi_{2}\right)I_{N},\\ D_{t}^{\alpha }A_{\beta }=\gamma_{ab}I_{N}-\left(d_{b}+\kappa \right)A_{\beta },\\ D_{t}^{\alpha }T_{\mu }=\kappa A_{\beta }-\left(d_{m}+\rho \right)T_{\mu }. \end{array}$$

We write the system as


$$D_{t}^{\alpha }X=\left(F-V\right)X,$$

with


$$F=\left(\begin{array}{ccc} 0 & \alpha_{\text{inf}}F_{N}^{*} & 0\\ \gamma_{ab} & 0 & 0\\ 0 & \kappa & 0 \end{array}\right),\;V=\left(\begin{array}{ccc} \gamma_{ab}+\phi_{2} & 0 & 0\\ 0 & d_{b}+\kappa & 0\\ 0 & 0 & d_{m}+\rho \end{array}\right).$$

Since *V* is diagonal, its inverse is


$$V^{-1}=\mathrm{diag}\text{​}({\left(\gamma_{ab}+\phi_{2}\right)}^{-1},\;{\left(d_{b}+\kappa \right)}^{-1},\;{\left(d_{m}+\rho \right)}^{-1}).$$

Hence the next-generation matrix is


$$K=FV^{-1}=\left(\begin{array}{ccc} 0 & \frac{\alpha_{\text{inf}}F_{N}^{*}}{d_{b}+\kappa } & 0\\ \frac{\gamma_{ab}}{\gamma_{ab}+\phi_{2}} & 0 & 0\\ 0 & \frac{\kappa }{d_{b}+\kappa } & 0 \end{array}\right).$$

The characteristic polynomial is


$$\det\left(K-\lambda I\right)=\lambda \left(-\lambda^{2}+\frac{\alpha_{\text{inf}}F_{N}^{*}}{d_{b}+\kappa }\cdot \frac{\gamma_{ab}}{\gamma_{ab}+\phi_{2}}\right).$$

Thus the eigenvalues are


$$\lambda_{1}=0,\;\lambda_{2,3}=\pm \sqrt{\frac{\alpha_{\text{inf}}F_{N}^{*}}{d_{b}+\kappa }\cdot \frac{\gamma_{ab}}{\gamma_{ab}+\phi_{2}}}.$$

The spectral radius of *K* (the largest eigenvalue in absolute value) is


$$R_{0}=\sqrt{\frac{\alpha_{\text{inf}}\gamma_{ab}}{\left(d_{b}+\kappa \right)\left(\gamma_{ab}+\phi_{2}\right)}\cdot \frac{\Pi_{N}}{\phi_{1}}}.$$

**Figure 1:**
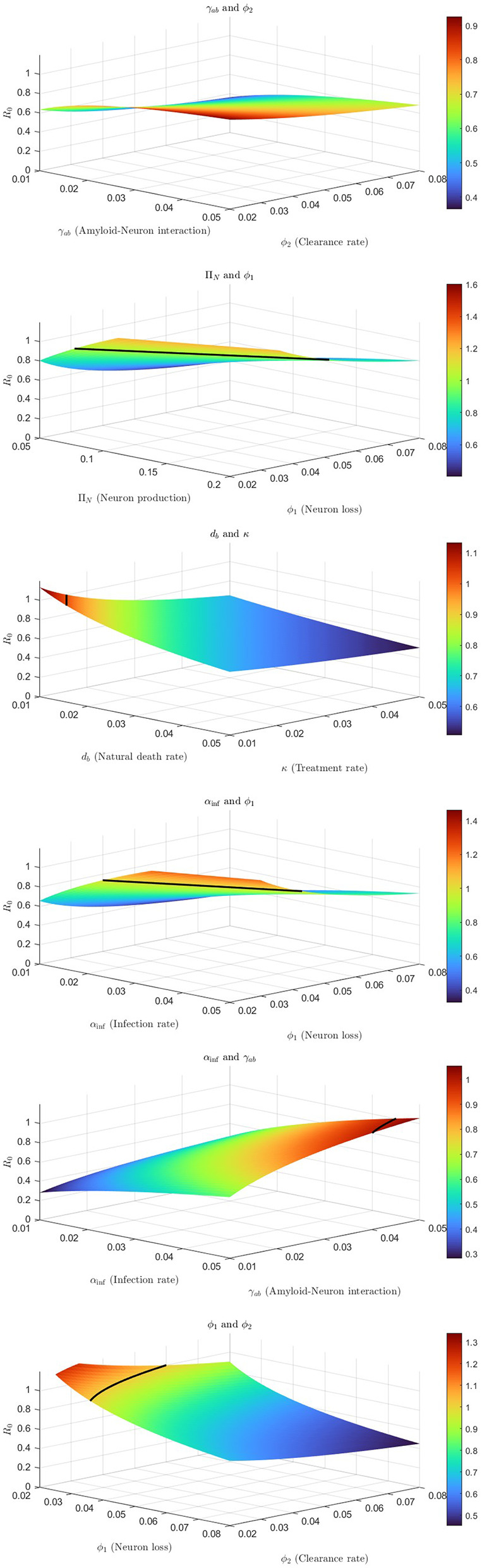
Three-dimensional sensitivity surfaces of the basic reproduction number *R*_0_ with respect to Alzheimer's disease model parameters, illustrating key parameter interactions, with black curves indicating the threshold *R*_0_ = 1.

Figure 1 shows how variations in key parameters influence the basic reproduction number *R*_0_, reflecting the system's sensitivity.

### 3.6 Iterative and stability analysis via Caputo operator

Theorem 3. Let (𝔉, |·|) be a Banach space and 𝔈: *𝔉* → *𝔉* be a mapping that satisfies:


$$\begin{array}{l} \|E_{m}-E_{n}\|\leq 𝔍\|X-E_{m}\|+\pi \|m-n\|, \end{array}$$ (39)

for all *m, n* ∈ 𝔉, where 0 ≤ 𝔍 and 0 ≤ π < 1. Then 𝔈 is Picard 𝔈-stable.

Theorem 4. Assume that 𝔈 is a self map with the following definition:


$$\begin{array}{l} E\left[F_{N_{\alpha }}\left(t\right)\right]=F_{N_{\alpha +1}}\left(t\right)=F_{N_{\alpha }}\left(0\right)\\ +L^{-1}\left[\frac{1}{E_{\sigma }}L\left\{\Pi_{N}+\rho T_{\mu_{\alpha }}-\alpha F_{N_{\alpha }}A_{\beta_{\alpha }}-\phi_{1}F_{N_{\alpha }}\right\}\right],\\ E\left[I_{N_{\alpha }}\left(t\right)\right]=I_{N_{\alpha +1}}\left(t\right)=I_{N_{\alpha }}\left(0\right)\\ +L^{-1}\left[\frac{1}{E_{\sigma }}L\left\{\alpha F_{N_{\alpha }}A_{\beta_{\alpha }}-\beta_{1}I_{N_{\alpha }}M_{\rho_{\alpha }}-\left(\gamma +\phi_{2}\right)I_{N_{\alpha }}\right\}\right],\\ E\left[A_{\beta_{\alpha }}\left(t\right)\right]=A_{\beta_{\alpha +1}}\left(t\right)=A_{\beta_{\alpha }}\left(0\right)\\ +L^{-1}\left[\frac{1}{E_{\sigma }}L\left\{\gamma I_{N_{\alpha }}-\beta_{2}A_{\beta_{\alpha }}M_{\rho_{\alpha }}-\left(d_{\beta }+\kappa \right)A_{\beta_{\alpha }}\right\}\right],\\ E\left[T_{\mu_{\alpha }}\left(t\right)\right]=T_{\mu_{\alpha +1}}\left(t\right)=T_{\mu_{\alpha }}\left(0\right)\\ +L^{-1}\left[\frac{1}{E_{\sigma }}L\left\{\kappa A_{\beta_{\alpha }}-\beta_{3}T_{\mu_{\alpha }}M_{\rho_{\alpha }}-\left(d_{\mu }+\rho \right)T_{\mu_{\alpha }}\right\}\right],\\ E\left[M_{\rho_{\alpha }}\left(t\right)\right]=M_{\rho_{\alpha +1}}\left(t\right)=M_{\rho_{\alpha }}\left(0\right)\\ +L^{-1}\left[\frac{1}{E_{\sigma }}L\left\{\left(\beta_{1}I_{N_{\alpha }}+\beta_{2}A_{\beta_{\alpha }}+\beta_{3}T_{\mu_{\alpha }}\right)M_{\rho_{\alpha }}-\phi_{3}M_{\rho_{\alpha }}\right\}\right]. \end{array}$$ (40)

The iteration is 𝔄-stable in *L*^1^(α, β) if the following criteria are met:


$$\begin{array}{l} \left(1-\rho w_{1}(\varphi )-\alpha \left(K_{1}+K_{2}\right)w_{2}(\varphi )-\phi_{1}w_{3}(\varphi )\right)<1,\\ (1+\alpha \left(K_{1}+K_{2}\right)w_{2}(\varphi )-\beta_{1}\left(K_{3}+K_{4}\right)w_{4}(\varphi )-\left(\gamma +\phi_{2}\right)\\ w_{5}(\varphi ))<1,\\ \left(1+\gamma w_{6}(\varphi )-\beta_{2}\left(K_{5}+K_{6}\right)w_{7}-\left(d_{\beta }+\kappa \right)w_{8}(\varphi )\right)<1,\\ \left(1+\kappa w_{8}(\varphi )-\beta_{3}\left(K_{7}+K_{8}\right)w_{9}-\left(d_{\mu }+\rho \right)w_{10}(\varphi )\right)<1,\\ (1+\beta_{1}\left(K_{3}+K_{4}\right)\left(w_{4}(\varphi )+\beta_{2}\left(K_{5}+K_{6}\right)w_{7}\right)+\beta_{3}\left(K_{7}+K_{8}\right)\\ w_{9}-\phi_{3}w_{11}(\varphi ))<1. \end{array}$$ (41)

*Proof*. We examine the following for (α, β) ∈ ℕ × ℕ in order to show that 𝔈 has a fixed point:


$$\begin{array}{l} E\left[F_{N_{\beta }}\left(t\right)\right]-E\left[F_{N_{\alpha }}\left(t\right)\right]=F_{N_{\beta }}\left(t\right)-F_{N_{\alpha }}\left(t\right)\\ +L^{-1}\left[\frac{1}{E_{\sigma }}L\left\{\Pi_{N}+\rho T_{\mu_{\beta }}-\alpha F_{N_{\beta }}A_{\beta_{\beta }}-\phi_{1}F_{N_{\beta }}\right\}\right]\\ -L^{-1}\left[\frac{1}{E_{\sigma }}L\left\{\Pi_{N}+\rho F_{N_{\alpha }}A_{\beta_{\alpha }}-\phi_{1}F_{N_{\alpha }}\right\}\right]. \end{array}$$ (42)

$$\begin{array}{l} E\left[I_{N_{\beta }}\left(t\right)\right]-E\left[I_{N_{\alpha }}\left(t\right)\right]=I_{N_{\beta }}\left(t\right)-I_{N_{\alpha }}\left(t\right)\\ +L^{-1}\left[\frac{1}{E_{\sigma }}L\left\{\alpha F_{N_{\beta }}A_{\beta_{\beta }}-\beta_{1}I_{N_{\beta }}M_{\rho_{\beta }}-\left(\gamma +\phi_{2}\right)I_{N_{\beta }}\right\}\right]\\ -L^{-1}\left[\frac{1}{E_{\sigma }}L\left\{\alpha F_{N_{\alpha }}A_{\beta_{\alpha }}-\beta_{1}I_{N_{\alpha }}M_{\rho_{\alpha }}-\left(\gamma +\phi_{2}\right)I_{N_{\alpha }}\right\}\right].\\ E\left[A_{\beta_{\beta }}\left(t\right)\right]-E\left[A_{\beta_{\alpha }}\left(t\right)\right]=A_{\beta_{\beta }}\left(t\right)-A_{\beta_{\alpha }}\left(t\right)\\ +L^{-1}\left[\frac{1}{E_{\sigma }}L\left\{\gamma I_{N_{\beta }}-\beta_{2}A_{\beta_{\beta }}M_{\rho_{\beta }}-\left(d_{\beta }+\kappa \right)A_{\beta_{\beta }}\right\}\right]\\ -L^{-1}\left[\frac{1}{E_{\sigma }}L\left\{\gamma I_{N_{\alpha }}-\beta_{2}A_{\beta_{\alpha }}M_{\rho_{\alpha }}-\left(d_{\beta }+\kappa \right)A_{\beta_{\alpha }}\right\}\right]. \end{array}$$ (43)

$$\begin{array}{l} E\left[T_{\mu_{\beta }}\left(t\right)\right]-E\left[T_{\mu_{\alpha }}\left(t\right)\right]=T_{\mu_{\beta }}\left(t\right)-T_{\mu_{\alpha }}\left(t\right)\\ +L^{-1}\left[\frac{1}{E_{\sigma }}L\left\{\kappa A_{\beta_{\beta }}-\beta_{3}T_{\mu_{\beta }}M_{\rho_{\beta }}-\left(d_{\mu }+\rho \right)T_{\mu_{\beta }}\right\}\right]\\ -L^{-1}\left[\frac{1}{E_{\sigma }}L\left\{\kappa A_{\beta_{\alpha }}-\beta_{3}T_{\mu_{\alpha }}M_{\rho_{\alpha }}-\left(d_{\mu }+\rho \right)T_{\mu_{\alpha }}\right\}\right]. \end{array}$$ (44)

$$\begin{array}{l} E\left[M_{\rho_{\beta }}\left(t\right)\right]-E\left[M_{\rho_{\alpha }}\left(t\right)\right]=M_{\rho_{\beta }}\left(t\right)-M_{\rho_{\alpha }}\left(t\right)\\ +L^{-1}\left[\frac{1}{E_{\sigma }}L\left\{\left(\beta_{1}I_{N_{\beta }}+\beta_{2}A_{\beta_{\beta }}+\beta_{3}T_{\mu_{\beta }}\right)M_{\rho_{\beta }}-\phi_{3}M_{\rho_{\beta }}\right\}\right]\\ -L^{-1}\left[\frac{1}{E_{\sigma }}L\left\{\left(\beta_{1}I_{N_{\alpha }}+\beta_{2}A_{\beta_{\alpha }}+\beta_{3}T_{\mu_{\alpha }}\right)M_{\rho_{\alpha }}-\phi_{3}M_{\rho_{\alpha }}\right\}\right]. \end{array}$$ (45)

By calculating the norm of both sides of the above equations, we obtain:


$$\begin{array}{l} \left\|E\left[F_{N_{\beta }}\right]-E\left[F_{N_{\alpha }}\right]\right\|\leq \left\|F_{N_{\beta }}-F_{N_{\alpha }}\right\|\\ +{ℒ}^{-1}[\frac{1}{E_{\sigma }}ℒ\{\left\|\rho \left(T_{\mu_{\beta }}-T_{\mu_{\alpha }}\right)\right\|\\ -\left\|\alpha F_{N_{\beta }}\left(A_{\beta_{\beta }}-A_{\beta_{\alpha }}\right)\right\|+\left\|\alpha A_{\beta_{\alpha }}\left(F_{N_{\beta }}-F_{N_{\alpha }}\right)\right\|\\ +\left\|-\phi_{1}\left(F_{N_{\beta }}-F_{N_{\alpha }}\right)\right\|+\cdots . \end{array}$$ (46)

$$\begin{array}{l} \left\|E\left[I_{N_{\beta }}(t)\right]-E\left[I_{N_{\alpha }}(t)\right]\right\|\leq \left\|I_{N_{\beta }}(t)-I_{N_{\alpha }}(t)\right\|\\ +{ℒ}^{-1}[\frac{1}{E_{\sigma }}ℒ\{\left\|-\left(\gamma +\phi_{2}\right)\left(I_{N_{\beta }}-I_{N_{\alpha }}\right)\right\|\\ +\left\|\alpha F_{N_{\beta }}\left(A_{\beta_{\beta }}-A_{\beta_{\alpha }}\right)\right\|+\left\|\alpha A_{\beta_{\alpha }}\left(F_{N_{\beta }}-F_{N_{\alpha }}\right)\right\|+\cdots . \end{array}$$ (47)

$$\begin{array}{l} \left\|E\left[A_{\beta_{\beta }}(t)\right]-E\left[A_{\beta_{\alpha }}(t)\right]\right\|\leq \left\|A_{\beta_{\beta }}(t)-A_{\beta_{\alpha }}(t)\right\|\\ +{ℒ}^{-1}[\frac{1}{E_{\sigma }}ℒ\{\left\|\gamma \left(I_{N_{\beta }}-I_{N_{\alpha }}\right)\right\|\\ -\left\|\beta_{2}A_{\beta_{\beta }}\left(M_{\rho_{\beta }}-M_{\rho_{\alpha }}\right)\right\|+\left\|\beta_{2}M_{\rho_{\alpha }}\left(A_{\beta_{\beta }}-A_{\beta_{\alpha }}\right)\right\|+\cdots . \end{array}$$ (48)

$$\begin{array}{l} \left\|E\left[T_{\mu_{\beta }}(t)\right]-E\left[T_{\mu_{\alpha }}(t)\right]\right\|\leq \left\|T_{\mu_{\beta }}(t)-T_{\mu_{\alpha }}(t)\right\|\\ +{ℒ}^{-1}[\frac{1}{E_{\sigma }}ℒ\{\left\|\kappa \left(A_{\beta_{\beta }}-A_{\beta_{\alpha }}\right)\right\|\\ -\left\|\beta_{3}T_{\mu_{\beta }}\left(M_{\rho_{\beta }}-M_{\rho_{\alpha }}\right)\right\|+\left\|\beta_{3}M_{\rho_{\alpha }}\left(T_{\mu_{\beta }}-T_{\mu_{\alpha }}\right)\right\|+\cdots . \end{array}$$ (49)

$$\begin{array}{l} \left\|E\left[M_{\rho_{\beta }}(t)\right]-E\left[M_{\rho_{\alpha }}(t)\right]\right\|\leq \left\|M_{\rho_{\beta }}(t)-M_{\rho_{\alpha }}(t)\right\|\\ +{ℒ}^{-1}[\frac{1}{E_{\sigma }}ℒ\{\|(\beta_{1}\left(I_{N_{\beta }}-I_{N_{\alpha }}\right)\|+\left\|\beta_{2}\left(A_{\beta_{\beta }}-A_{\beta_{\alpha }}\right)\right\|\\ \;+\left\|\beta_{3}\left(T_{\mu_{\beta }}-T_{\mu_{\alpha }}\right)\left(M_{\rho_{\beta }}-M_{\rho_{\alpha }}\right)\right\|+\cdots . \end{array}$$ (50)

Let,


$$\begin{array}{l} \left\|F_{N_{\beta }}\left(t\right)-F_{N_{\alpha }}\left(t\right)\|≅\|I_{N_{\beta }}\left(t\right)-I_{N_{\alpha }}\left(t\right)\|≅\|A_{\beta_{\beta }}\left(t\right)-A_{\beta_{\alpha }}\left(t\right)\right\|\\ ≅\|T_{\mu_{\beta }}\left(t\right)-T_{\mu_{\alpha }}\left(t\right)\|≅\|M_{\rho_{\beta }}\left(t\right)-M_{\rho_{\alpha }}\left(t\right)\|. \end{array}$$ (51)

To get the following relation:


$$\begin{array}{l} \left\|E\left[F_{N_{\beta }}\left(t\right)\right]-E\left[F_{N_{\alpha }}\left(t\right)\right]\right\|\\ \leq \left(1-\rho w_{1}\left(\varphi \right)-\alpha \left(K_{1}+K_{2}\right)w_{2}\left(\varphi \right)-\phi_{1}w_{3}\left(\varphi \right)\right)\\ \|F_{N_{\beta }}-F_{N_{\alpha }}\|, \end{array}$$ (52)

$$\begin{array}{l} \left\|E\left[I_{N_{\beta }}(t)\right]-E\left[I_{N_{\alpha }}(t)\right]\right\|\leq (1+\alpha \left(K_{1}+K_{2}\right)w_{2}(\varphi )\\ -\beta_{1}\left(K_{3}+K_{4}\right)w_{4}(\varphi )-\left(\gamma +\phi_{2}\right)w_{5}(\varphi ))\\ \left\|I_{N_{\beta }}-I_{N_{\alpha }}\right\|, \end{array}$$ (53)

$$\begin{array}{l} \left\|E\left[A_{\beta_{\beta }}\left(t\right)\right]-E\left[A_{\beta_{\alpha }}\left(t\right)\right]\right\|\\ \leq \left(1+\gamma w_{6}\left(\varphi \right)-\beta_{2}\left(K_{5}+K_{6}\right)w_{7}-\left(d_{\beta }+\kappa \right)w_{8}\left(\varphi \right)\right)\\ \|A_{\beta_{\beta }}-A_{\beta_{\alpha }}\|, \end{array}$$ (54)

$$\begin{array}{l} \left\|E\left[T_{\mu_{\beta }}(t)\right]-E\left[T_{\mu_{\alpha }}(t)\right]\right\|\leq (1+\kappa w_{8}(\varphi )\\ -\beta_{3}\left(K_{7}+K_{8}\right)w_{9}-\left(d_{\mu }+\rho \right)w_{10}(\varphi ))\left\|T_{\mu_{\beta }}-T_{\mu_{\alpha }}\right\|, \end{array}$$ (55)

$$\begin{array}{l} \left\|E\text{​}\left[M_{\rho_{\beta }}\right]-E\text{​}\left[M_{\rho_{\alpha }}\right]\right\|\leq (1+\beta_{1}\text{​}\left(K_{3}+K_{4}\right))(w_{4}(\varphi )\\ +\beta_{2}\text{​}\left(K_{5}+K_{6}\right)w_{7}+\beta_{3}\text{​}\left(K_{7}+K_{8}\right)w_{9}\\ -\phi_{3}w_{11}(\varphi ))\left\|M_{\rho_{\beta }}-M_{\rho_{\alpha }}\right\|. \end{array}$$ (56)

Also, the convergent sequences *F*_*N*_β__, *I*_*N*_β__, *A*_β_β__, *T*_μ_β__ and *M*_ρ_β__ are bounded. Then we obtain positive constants *K*_1_, *K*_2_, *K*_3_, *K*_4_ and *K*_5_ for all *t* such that


$$\begin{array}{c} \|F_{N_{\alpha }}\|<K_{1},\;\|I_{N_{\alpha }}\|<K_{2},\;\|A_{\beta_{\alpha }}\|<K_{3},\;\|T_{\mu_{\alpha }}\|<K_{4},\\ \;\|M_{\rho_{\alpha }}\|<K_{5},\;\left(\beta ,\alpha \right)\in \mathbb{N}\times \mathbb{N}. \end{array}$$ (57)

Then from Equations 56–60, 61, we get


$$\begin{array}{c} \left\|E\left[F_{N_{\beta }}(t)\right]-E\left[F_{N_{\alpha }}(t)\right]\right\|\leq (1-\rho w_{1}(\varphi )-\alpha \left(K_{1}+K_{2}\right)\\ w_{2}(\varphi )-\phi_{1}w_{3}(\varphi ))\left\|F_{N_{\beta }}-F_{N_{\alpha }}\right\|. \end{array}$$ (58)

Where 𝔴_1_, 𝔴_2_, 𝔴_3_, 𝔴_4_ and 𝔴_5_ are functions of $L^{-1}\left\{\frac{1}{E_{\sigma }}L\right\}$.

Similarly,


$$\begin{array}{l} \left\|E\left[I_{N_{\beta }}\right]-E\left[I_{N_{\alpha }}\right]\right\|\leq (1+\alpha \left(K_{1}+K_{2}\right)w_{2}(\varphi )-\beta_{1}\left(K_{3}+K_{4}\right)\\ w_{4}(\varphi )-\left(\gamma +\phi_{2}\right)w_{5}(\varphi ))\left\|I_{N_{\beta }}-I_{N_{\alpha }}\right\|,\\ \left\|E\left[A_{\beta_{\beta }}(t)\right]-E\left[A_{\beta_{\alpha }}(t)\right]\right\|\leq (1+\gamma w_{6}(\varphi )-\beta_{2}\left(K_{5}+K_{6}\right)w_{7}\\ -\left(d_{\beta }+\kappa \right)w_{8}(\varphi ))\left\|A_{\beta_{\beta }}-A_{\beta_{\alpha }}\right\|,\\ \left\|E\left[T_{\mu_{\beta }}(t)\right]-E\left[T_{\mu_{\alpha }}(t)\right]\right\|\leq (1+\kappa w_{8}(\varphi )-\beta_{3}\left(K_{7}+K_{8}\right)w_{9}\\ -\left(d_{\mu }+\rho \right)w_{10}(\varphi ))\left\|T_{\mu_{\beta }}-T_{\mu_{\alpha }}\right\|,\\ \left\|E\text{​}\left[M_{\rho_{\beta }}\right]-E\text{​}\left[M_{\rho_{\alpha }}\right]\right\|\leq (1+\beta_{1}\text{​}\left(K_{3}+K_{4}\right))(w_{4}(\varphi )\\ +\beta_{2}\text{​}\left(K_{5}+K_{6}\right)w_{7}+\beta_{3}\text{​}\left(K_{7}+K_{8}\right)w_{9}\\ -\phi_{3}w_{11}(\varphi ))\left\|M_{\rho_{\beta }}-M_{\rho_{\alpha }}\right\|. \end{array}$$ (59)

Where


$$\begin{array}{l} \left(1-\rho w_{1}(\varphi )-\alpha \left(K_{1}+K_{2}\right)w_{2}(\varphi )-\phi_{1}w_{3}(\varphi )\right)<1,\\ (1+\alpha \left(K_{1}+K_{2}\right)w_{2}(\varphi )-\beta_{1}\left(K_{3}+K_{4}\right)w_{4}(\varphi )-\left(\gamma +\phi_{2}\right)\\ w_{5}(\varphi ))<1,\\ \left(1+\gamma w_{6}(\varphi )-\beta_{2}\left(K_{5}+K_{6}\right)w_{7}-\left(d_{\beta }+\kappa \right)w_{8}(\varphi )\right)<1,\\ \left(1+\kappa w_{8}(\varphi )-\beta_{3}\left(K_{7}+K_{8}\right)w_{9}-\left(d_{\mu }+\rho \right)w_{10}(\varphi )\right)<1,\\ (1+\beta_{1}\left(K_{3}+K_{4}\right)(w_{4}(\varphi )+\beta_{2}\left(K_{5}+K_{6}\right)w_{7}+\beta_{3}\left(K_{7}+K_{8}\right)\\ w_{9}-\phi_{3}w_{11}(\varphi )))<1. \end{array}$$ (60)

As 𝔈 has a fixed point and using Equations 62, 63, let


$$\begin{array}{l} \pi =\left(0,0,0,0,0\right), \end{array}$$ (61)

$$ℑ=\{\begin{array}{l} \left(1-\rho w_{1}(\varphi )-\alpha \left(K_{1}+K_{2}\right)w_{2}(\varphi )-\phi_{1}w_{3}(\varphi )\right),\\ (1+\alpha \left(K_{1}+K_{2}\right)w_{2}(\varphi )-\beta_{1}\left(K_{3}+K_{4}\right)w_{4}(\varphi )\\ -\left(\gamma +\phi_{2}\right)w_{5}(\varphi )),\\ \left(1+\gamma w_{6}(\varphi )-\beta_{2}\left(K_{5}+K_{6}\right)w_{7}-\left(d_{\beta }+\kappa \right)w_{8}(\varphi )\right),\\ \left(1+\kappa w_{8}(\varphi )-\beta_{3}\left(K_{7}+K_{8}\right)w_{9}-\left(d_{\mu }+\rho \right)w_{10}(\varphi )\right),\\ (1+\beta_{1}\left(K_{3}+K_{4}\right)(w_{4}(\varphi )+\beta_{2}\left(K_{5}+K_{6}\right)w_{7}\\ +\beta_{3}\left(K_{7}+K_{8}\right)w_{9}-\phi_{3}w_{11})). \end{array}$$ (62)

This completes the proof.

### 3.7 Local stability analysis

Definition 3.1. The Hartman-Grobman theorem states that if the linearization of the equations produces no zero or imaginary eigenvalues, then there is a continuous function with a continuous inverse in the region of this point into ℝ^*n*^.

The Jacobian matrix of the model (*F*_*N*_, *I*_*N*_, *A*_β_, *T*_μ_) is given by:


$$\left[\begin{array}{cccc} -\phi_{1} & 0 & \frac{-\alpha \Pi_{N}}{\phi_{1}} & \rho \\ 0 & -\gamma -\phi_{2} & \frac{\alpha \Pi_{N}}{\phi_{1}} & 0\\ 0 & \gamma & -d_{\beta }-\kappa & 0\\ 0 & 0 & \kappa & -d_{\mu }-\rho \end{array}\right]$$

All of the eigenvalues, which are calculated using Maple software, were found to have negative real values after verification. Thus, the equilibrium point can be considered locally stable.

### 3.8 Global stability analysis using Lyapunov function

Theorem 5. Consider the Alzheimer's fractional order system with the endemic equilibrium


$$E^{*}=\left(F_{N}^{*},I_{N}^{*},A_{\beta }^{*},T_{\mu }^{*},M_{\rho }^{*}\right).$$

If the condition


$$\Upsilon_{1}<\Upsilon_{2},$$

holds, then the equilibrium E^*^ is globally asymptotically stable in the invariant region.

*Proof*. Assume the Volterra-type Lyapunov function:


$$\begin{array}{l} L=L_{1}\left(F_{N}-F_{N}^{*}-F_{N}^{*}\log\frac{F_{N}}{F_{N}^{*}}\right)+L_{2}\left(I_{N}-I_{N}^{*}-I_{N}^{*}\log\frac{I_{N}}{I_{N}^{*}}\right)\\ \;+L_{3}\left(A_{\beta }-A_{\beta }^{*}-A_{\beta }^{*}\log\frac{A_{\beta }}{A_{\beta }^{*}}\right)+L_{4}\left(T_{\mu }-T_{\mu }^{*}-T_{\mu }^{*}\log\frac{T_{\mu }}{T_{\mu }^{*}}\right)\\ \;+L_{5}\left(M_{\rho }-M_{\rho }^{*}-M_{\rho }^{*}\log\frac{M_{\rho }}{M_{\rho }^{*}}\right) \end{array}$$

Assuming the positive constants *L*_*i*_, *i* = 1, 2, 3, 4, 5 and replacing Equation 53 in the system, Lemma 1 can be applied.


DtσCL≤L1(FN−FN*FN)cDtσFN+L2(IN−IN*IN)cDtσIN         +L3(Aβ−Aβ*Aβ)cDtσAβ+L4(Tμ−Tμ*Tμ)cDtσTμ         +L5(Mρ−Mρ*Mρ)cDtσMρ.

Now, modify the preceding formula's derivative to obtain:


DtσCL≤L1(FN−FN*FN)(ΠN+ρTμ−αFNAβ−ϕ1FN)          +L2(IN−IN*IN)(αFNAβ−β1INMρ−(γ+ϕ2)IN)          +L3(Aβ−Aβ*Aβ)(γIN−β2AβMρ−(dβ+κ)Aβ)          +L4(Tμ−Tμ*Tμ)(κAβ−β3TμMρ−(dμ+ρ)Tμ)          +L5(Mρ−Mρ*Mρ)((β1IN+β2Aβ+β3Tμ)          Mρ−ϕ3Mρ).

Now, by replacing $F_{N}=F_{N}-F_{N}^{*},I_{N}=I_{N}-I_{N}^{*},A_{\beta }=A_{\beta }-A_{\beta }^{*},T_{\mu }=T_{\mu }-T_{\mu }^{*},M_{\rho }=M_{\rho }-M_{\rho }^{*}$, we obtain:


DtσCL≤L1(FN−FN*FN)(ΠN+ρ(Tμ−Tμ*)−α(FN−FN*)          (Aβ−Aβ*)−ϕ1(FN−FN*))          +L2(IN−IN*IN)(α(FN−FN*)(Aβ−Aβ*)−β1(IN−IN*)           (Mρ−Mρ*)−(γ+ϕ2)(IN−IN*))          +L3(Aβ−Aβ*Aβ)(γ(IN−IN*)−β2(Aβ−Aβ*)           (Mρ−Mρ*)−(dβ+κ)(Aβ−Aβ*))           +L4(Tμ−Tμ*Tμ)(κ(Aβ−Aβ*)−β3(Tμ−Tμ*)           (Mρ−Mρ*)−(dμ+ρ)(Tμ−Tμ*))           +L5(Mρ−Mρ*Mρ)(β1(IN−IN*)+β2(Aβ−Aβ*)           +β3(Tμ−Tμ*))(Mρ−Mρ*)−ϕ3(Mρ−Mρ*).

Now, assume *L*_1_ = *L*_2_ = *L*_3_ = *L*_4_ = *L*_5_ = 1 and by rearranging the equation above, we obtain:


DtσCL≤ΠN−ΠNFN*FN+ρTμ−ρTμ*−ρTμFN*FN                        +ρTμ*FN*FN−αAβ(FN−FN*)2FN+αAβ*(FN−FN*)2FN                        −ϕ1(FN−FN*)2FN+αFNAβ−αFNAβ*−αFN*Aβ                        +αFN*Aβ*−αFNAβIN*IN+αFNAβ*IN*IN                        +αFN*AβIN*IN−αFN*Aβ*IN*IN−β1Mρ(IN−IN*)2IN                        +β1Mρ*(IN−IN*)2IN                        −(γ+ϕ2)(IN−IN*)2IN+γIN−γIN*−γINAβ*Aβ                        +γIN*Aβ*Aβ−β2Mρ(Aβ−Aβ*)2Aβ                        +β2Mρ*(Aβ−Aβ*)2Aβ−(dβ+κ)(Aβ−Aβ*)2Aβ                        +κAβ−κAβ*−κAβTμ*Tμ                        +κAβ*Tμ*Tμ−β3Mρ(Tμ−Tμ*)2Tμ+β3Mρ*(Tμ−Tμ*)2Tμ                       −(dμ+ρ)(Tμ−Tμ*)2Tμ                      +β1IN(Mρ−Mρ*)2Mρ−β1IN*(Mρ−Mρ*)2Mρ                      +β2Aβ(Mρ−Mρ*)2Mρ−β2Aβ*(Mρ−Mρ*)2Mρ                      +β3Tμ(Mρ−Mρ*)2Mρ−β3Tμ*(Mρ−Mρ*)2Mρ−ϕ3                       (Mρ−Mρ*)2Mρ.

By using the following assumption,


DtσCL≤Υ1−Υ2

where


$$\begin{array}{l} \Upsilon_{1}=\Pi_{N}+\rho T_{\mu }+\rho T_{\mu }^{*}\frac{F_{N}^{*}}{F_{N}}+\alpha A_{\beta }^{*}\frac{{\left(F_{N}-F_{N}^{*}\right)}^{2}}{F_{N}}+\alpha F_{N}A_{\beta }\\ \;+\alpha F_{N}^{*}A_{\beta }^{*}+\alpha F_{N}A_{\beta }^{*}\frac{I_{N}^{*}}{I_{N}}\\ \;+\alpha F_{N}^{*}A_{\beta }\frac{I_{N}^{*}}{I_{N}}+\beta_{1}M_{\rho }^{*}\frac{{\left(I_{N}-I_{N}^{*}\right)}^{2}}{I_{N}}+\phi_{2}\frac{{\left(I_{N}-I_{N}^{*}\right)}^{2}}{I_{N}}\\ \;+\gamma I_{N}+\gamma I_{N}^{*}\frac{A_{\beta }^{*}}{A_{\beta }}+\beta_{2}M_{\rho }\frac{{\left(A_{\beta }-A_{\beta }^{*}\right)}^{2}}{A_{\beta }}+\kappa A_{\beta }\\ \;+\kappa A_{\beta }^{*}\frac{T_{\mu }^{*}}{T_{\mu }}+\beta_{3}M_{\rho }^{*}\frac{{\left(T_{\mu }-T_{\mu }^{*}\right)}^{2}}{T_{\mu }}+\beta_{1}I_{N}\frac{{\left(M_{\rho }-M_{\rho }^{*}\right)}^{2}}{M_{\rho }}\\ \;+\beta_{2}A_{\beta }\frac{{\left(M_{\rho }-M_{\rho }^{*}\right)}^{2}}{M_{\rho }}+\beta_{3}T_{\mu }\frac{{\left(M_{\rho }-M_{\rho }^{*}\right)}^{2}}{M_{\rho }}, \end{array}$$

and


$$\begin{array}{l} \Upsilon_{2}=\Pi_{N}\frac{F_{N}^{*}}{F_{N}}+\rho T_{\mu }^{*}+\rho T_{\mu }\frac{F_{N}^{*}}{F_{N}}+\alpha A_{\beta }\frac{{\left(F_{N}-F_{N}^{*}\right)}^{2}}{F_{N}}\\ \;+\phi_{1}\frac{{\left(F_{N}-F_{N}^{*}\right)}^{2}}{F_{N}}+\alpha F_{N}A_{\beta }^{*}+\alpha F_{N}^{*}A_{\beta }\\ \;+\alpha F_{N}A_{\beta }\frac{I_{N}^{*}}{I_{N}}+\alpha F_{N}^{*}A_{\beta }^{*}\frac{I_{N}^{*}}{I_{N}}+\beta_{1}M_{\rho }\frac{{\left(I_{N}-I_{N}^{*}\right)}^{2}}{I_{N}}+\left(\gamma +\phi_{2}\right)\\ \;\frac{{\left(I_{N}-I_{N}^{*}\right)}^{2}}{I_{N}}+\gamma I_{N}^{*}\\ \;+\gamma I_{N}\frac{A_{\beta }^{*}}{A_{\beta }}+\beta_{2}M_{\rho }\frac{{\left(A_{\beta }-A_{\beta }^{*}\right)}^{2}}{A_{\beta }}+\left(d_{\beta }+\kappa \right)\frac{{\left(A_{\beta }-A_{\beta }^{*}\right)}^{2}}{A_{\beta }}\\ \;+\kappa A_{\beta }^{*}+\kappa A_{\beta }\frac{T_{\mu }^{*}}{T_{\mu }}\\ \;+\beta_{3}M_{\rho }\frac{{\left(T_{\mu }-T_{\mu }^{*}\right)}^{2}}{T_{\mu }}+\left(d_{\mu }+\rho \right)\frac{{\left(T_{\mu }-T_{\mu }^{*}\right)}^{2}}{T_{\mu }}\\ \;+\beta_{1}I_{N}^{*}\frac{{\left(M_{\rho }-M_{\rho }^{*}\right)}^{2}}{M_{\rho }}+\beta_{2}A_{\beta }^{*}\frac{{\left(M_{\rho }-M_{\rho }^{*}\right)}^{2}}{M_{\rho }}\\ \;+\beta_{3}T_{\mu }^{*}\frac{{\left(M_{\rho }-M_{\rho }^{*}\right)}^{2}}{M_{\rho }}+\phi_{3}\frac{{\left(M_{\rho }-M_{\rho }^{*}\right)}^{2}}{M_{\rho }}. \end{array}$$

If


$$\Upsilon_{1}<\Upsilon_{2}arrow^{c}D_{t}^{\sigma }L<0,$$

now, let $F_{N}=F_{N}^{*},I_{N}=I_{N}^{*},A_{\beta }=A_{\beta }^{*},T_{\mu }=T_{\mu }^{*},M_{\rho }=M_{\rho }^{*}$, then it can be concluded that $E^{*}=\left(F_{N}^{*},I_{N}^{*},A_{\beta }^{*},T_{\mu }^{*},M_{\rho }^{*}\right)$ is the compact invariant set in


$$\left\{\left(F_{N}^{*},I_{N}^{*},A_{\beta }^{*},T_{\mu }^{*},M_{\rho }^{*}\right)\in \Delta {:}^{c}D_{t}^{\sigma }L=0\right\}.$$

Hence, if Υ_1_ < Υ_2_, then the equilibrium points E^*^ are globally asymptotically stable in the invariant region.

### 3.9 Chaos stabilizing system

Utilizing the points of equilibrium, the linear output technique can be used to stabilize the suggested system (Equation 9), which is considered as a controlled-design fractional-order system (Nisar et al., 2024a,b).

Let ω_1_, ω_2_, ω_3_, ω_4_ and ω_5_ be the controlled parameters, while $F_{N}^{*},I_{N}^{*},A_{\beta }^{*}$ and $T_{\mu }^{*}$ are the proposed model equilibrium points and a Jacobian matrix can be constructed to be as:


$$\begin{array}{l} \left[\begin{array}{cccc} -\phi_{1}-\omega_{1} & 0 & \frac{-\alpha \Pi_{N}}{\phi_{1}} & \rho \\ 0 & -\gamma -\phi_{2}-\omega_{2} & \frac{\alpha \Pi_{N}}{\phi_{1}} & 0\\ 0 & \gamma & -d_{\beta }-\kappa -\omega_{3} & 0\\ 0 & 0 & \kappa & -d_{\mu }-\rho -\omega_{4} \end{array}\right] \end{array}$$ (63)

Assuming ω_1_ = 1, ω_2_ = 2, ω_3_ = 3, ω_4_ = 4 and ω_5_ = 5, with the values of the parameters ϕ_1_ = 0.00003, α = 0.08, Π_*N*_ = 700, ρ = 0.025, γ = 0.00017, ϕ_2_ = 0.00019, *d*_β_ = 9.51, κ = 0.025, *d*_μ_ = 0.277.By using Maple software, the roots are:


$$\begin{array}{l} \lambda_{1}=-1.00003000000000,\;\lambda_{2}=-4.30200000000000,\\ \lambda_{3}=-25.8118817160782,\;\lambda_{4}=-11.3265217160782 \end{array}$$ (64)

Since, all of of the eigenvalues in Equation are real and negative numbers, the equilibrium points are asymptotically stable.

**Figure 2:**
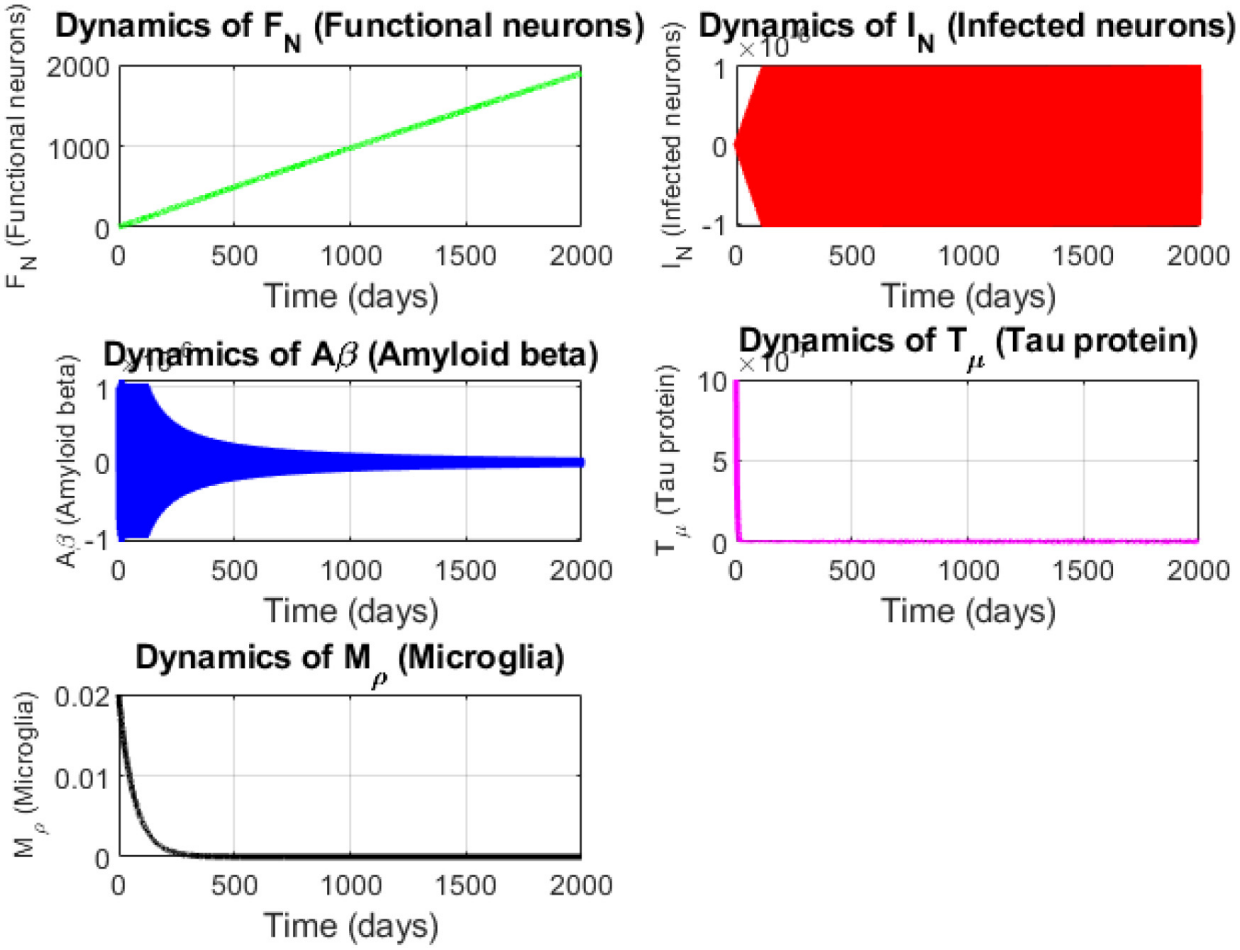
Dynamics of the Alzheimer's disease model compartments over 200 days. The plots show the temporal evolution of functional neurons (*F*_*N*_, green), infected neurons (*I*_*N*_, red), amyloid beta (*A*_β_, blue), tau protein (*T*_μ_, magenta) and microglia (*M*_ρ_, black). Functional neurons increase linearly due to a high production rate, infected neurons and amyloid beta exhibit unstable growth reflecting disease progression, tau protein stabilizes after an initial surge and microglia decline over time indicating immune exhaustion.

As illustrated in Figure 2, the dynamics of the Alzheimer's disease model compartments over 200 days are shown. The temporal evolution of functional neurons (*F*_*N*_), infected neurons (*I*_*N*_), amyloid beta (*A*_β_), tau protein (*T*_μ_) and microglia (*M*_ρ_) are depicted.

### 3.10 Logarithmic sensitivity analysis

We evaluate how small perturbations in key parameters affect model outcomes. The logarithmic sensitivity is defined as:


$$\begin{array}{l} S_{\log}\left(t\right)=\frac{\partial \ln\;y\left(t\right)}{\partial \ln\;p}\approx \frac{\ln\;y_{+}\left(t\right)-\ln\;y_{-}\left(t\right)}{2\ln\left(1+\delta \right)} \end{array}$$ (65)

where:

- *y*_+_(*t*): output with parameter *p*(1+δ)
- *y*_−_(*t*): output with parameter *p*(1−δ)
- δ = 0.1 (10% perturbation)

This formulation captures both the direction and magnitude of sensitivity in a normalized way.

## 4 Results interpretation

**Figure 3:**
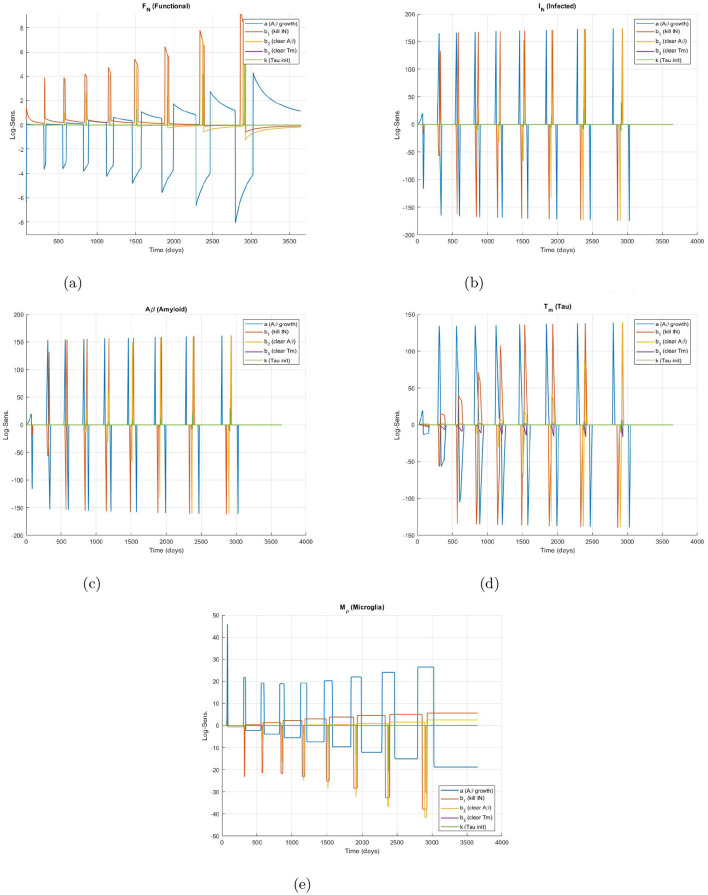
Time-dependent log-sensitivities of the Alzheimer model variables to ±10% perturbations in parameters. **(a)** Functional neurons (*F*_*N*_). **(b)** Infected neurons (*I*_*N*_). **(c)** Amyloid beta (*Aβ*). **(d)** Tau protein (*T*_*m*_). **(e)** Microglia (*M*_ρ_).

The time-dependent log-sensitivities of the Alzheimer model variables to ±10% perturbations in parameters are illustrated in Figure 3. The analysis reveals the following observations:

- **Functional neurons (*F*_*N*_)** (Figure 3a): High negative sensitivity to α (amyloid beta toxicity) indicating vulnerability to *A*_β_ buildup.
- **Infected neurons (*I*_*N*_)** (Figure 3b): Sensitive to both α and β_1_; infection rises with *A*_β_ and is controlled by microglia.
- **Amyloid beta (*A*_*β*_)** (Figure 3c): Strongly impacted by α, β_1_ and β_2_ reflecting production and clearance balance.
- **Tau protein (*T*_*μ*_)** (Figure 3d): Follows the dynamics of *A*_β_ and shows sensitivity to κ and β_3_.
- **Microglia (*M*_*ρ*_)** (Figure 3e): Fluctuates mildly, reflecting its stabilizing regulatory role.

Logarithmic sensitivity analysis using NSFD reveals nonlinear time varying dependence of disease progression on key biological parameters (see Table 2). This method guides strategic control interventions and enhances the understanding of neuro degenerative dynamics.

**Table 2:** Summary.

**Compartment**	**Dominant parameter(s)**	**Interpretation**
*F*_*N*_	α	*A*_β_ directly kills functional neurons
*I*_*N*_	α, β_1_	Infection rises with *A*_β_, falls with microglial clearance
*A*_β_	α, β_1_, β_2_	*A*_β_ is produced by infected neurons and cleared by microglia
*T*_μ_	α, κ	Tau protein increases following *A*_β_ buildup
*M*_ρ_	β_1_, β_2_, β_3_	Microglial levels reflect infection and toxic protein burden

### 4.1 Mapping model states to measurable biomarkers

The state variables of the proposed fractional-order model shows direct correspondence with clinically measurable biomarkers used in Alzheimer's disease diagnosis and progression assessment. The functional neuron population *F*_*N*_ is associated with neuroimaging-] derived indicators such as brain volume, cortical thickness and cognitive performance scores including MMSE and ADAS-Cog. Amyloid beta concentration *A*_β_ corresponds to cerebrospinal fluid (CSF) Aβ_42_ levels and amyloid PET imaging, while tau protein *T*_μ_ aligns with CSF phosphorylated tau biomarkers and tau PET tracers. The microglial compartment *M*_ρ_ reflects neuroinflammatory activity and can be linked to TSPO-PET imaging markers. This mapping enables the proposed framework to bridge mechanistic modeling with observable clinical data thus facilitating data-driven inference and model calibration.

### 4.2 Simulations

**Figure 4:**
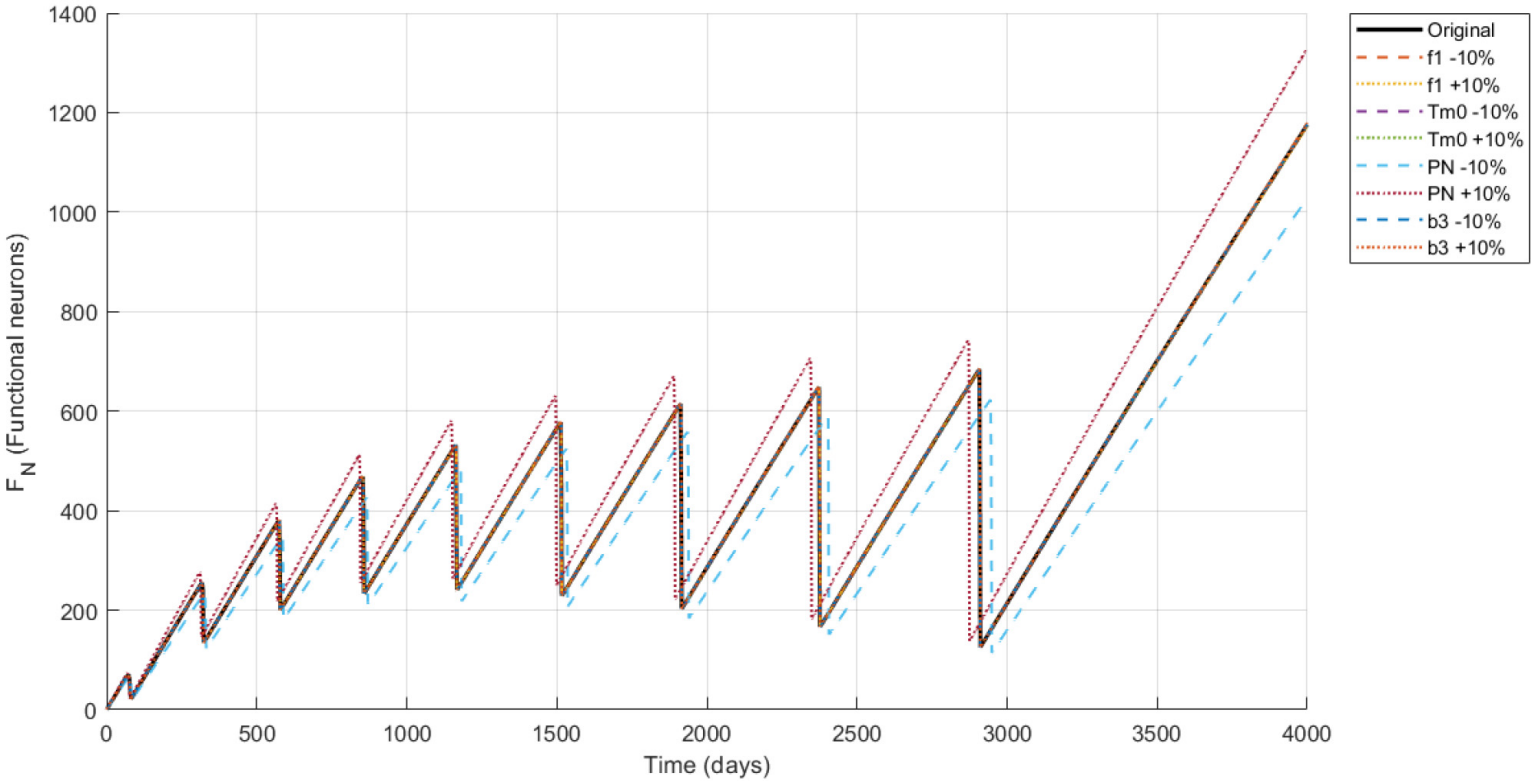
Dynamics of *F*_*N*_ (functional neurons) under ±10% parameter variation.

**Figure 5:**
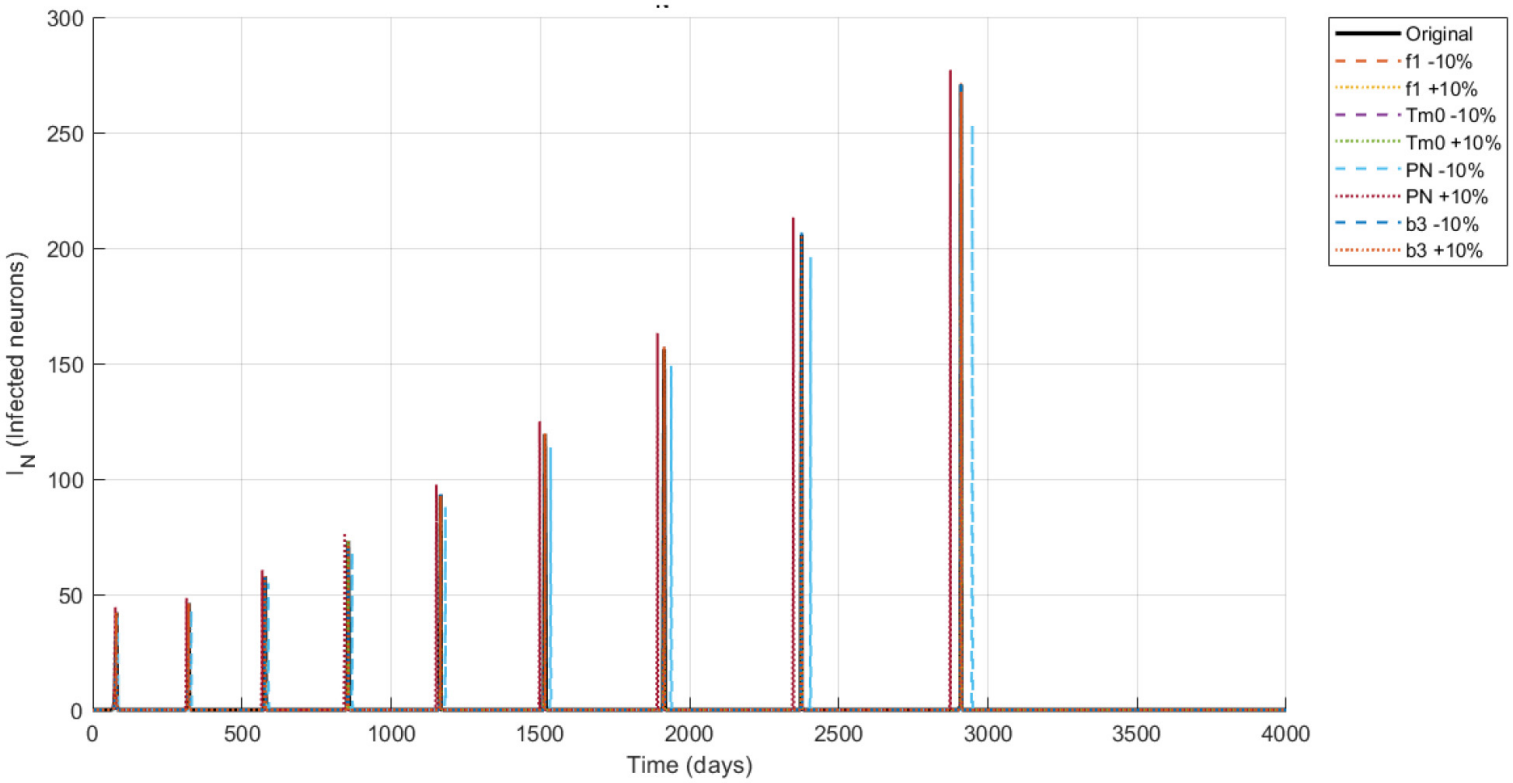
Dynamics of *I*_*N*_ (infected neurons) under ±10% parameter variation.

**Figure 6:**
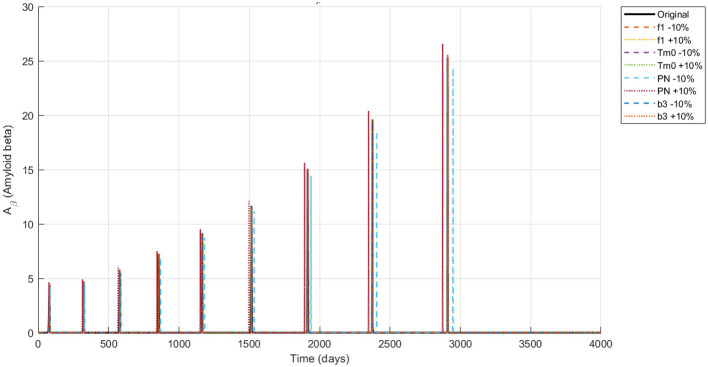
Dynamics of *A*_β_ (amyloid beta concentration) under ±10% parameter variation.

**Figure 7:**
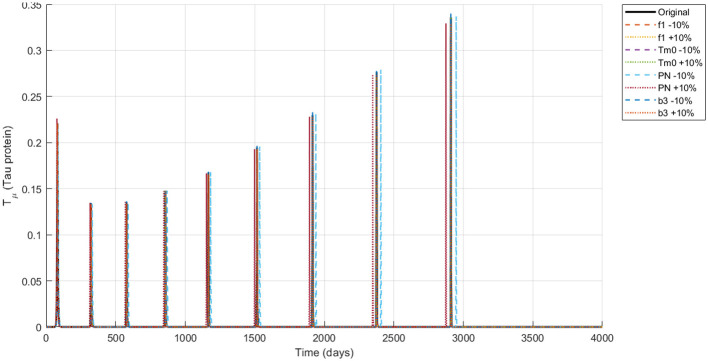
Dynamics of *T*_μ_ (tau protein concentration) under ±10% parameter variation.

**Figure 8:**
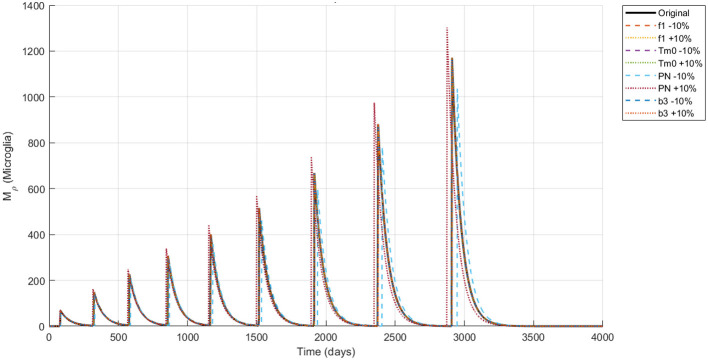
Dynamics of *M*_ρ_ (microglia concentration) under ±10% parameter variation.

The simulation of the Alzheimer's disease model using the NSFD scheme with ±10% parameter variations in neuronal infection rate, initial Tau protein concentration, Neuron production rate and the amyloid to tau conversion rate reveals biologically consistent dynamics across all compartments are shown in Figures 4–8. Functional neurons show a gradual decline interspersed with fluctuations, where increased neuronal production mitigates losses while higher infection rates accelerate degeneration. Infected neurons (*I*_*N*_) exhibit spike like growth patterns whose magnitude and frequency are strongly affected by neuronal infection rate and Neuron production rate suggesting that even small changes in infection or neurogenesis processes can significantly shift neuronal vulnerability. Amyloid beta (*A*_β_) demonstrates recurrent aggregation peaks, with the timing and amplitude of these cycles markedly influenced by all four parameters highlighting their central role in driving pathological progression. Tau protein (*T*_μ_) oscillations are particularly sensitive to the initial tau burden (*T*_*m*0_) and the amyloid tau interaction rate (*b*_3_), where larger values accelerate accumulation and intensify spikes. Microglia (*M*_ρ_) display fluctuating activation, with bursts modulated by both neuronal production and infection processes, reflecting their downstream immune response to amyloid and neuronal damage. Overall, these results indicate that modest variations in critical parameters substantially reshape the long term trajectories of all biomarkers reinforcing the model's ability to capture the delicate balance between neuronal survival protein aggregation and immune activation in Alzheimer's disease.

**Figure 9:**
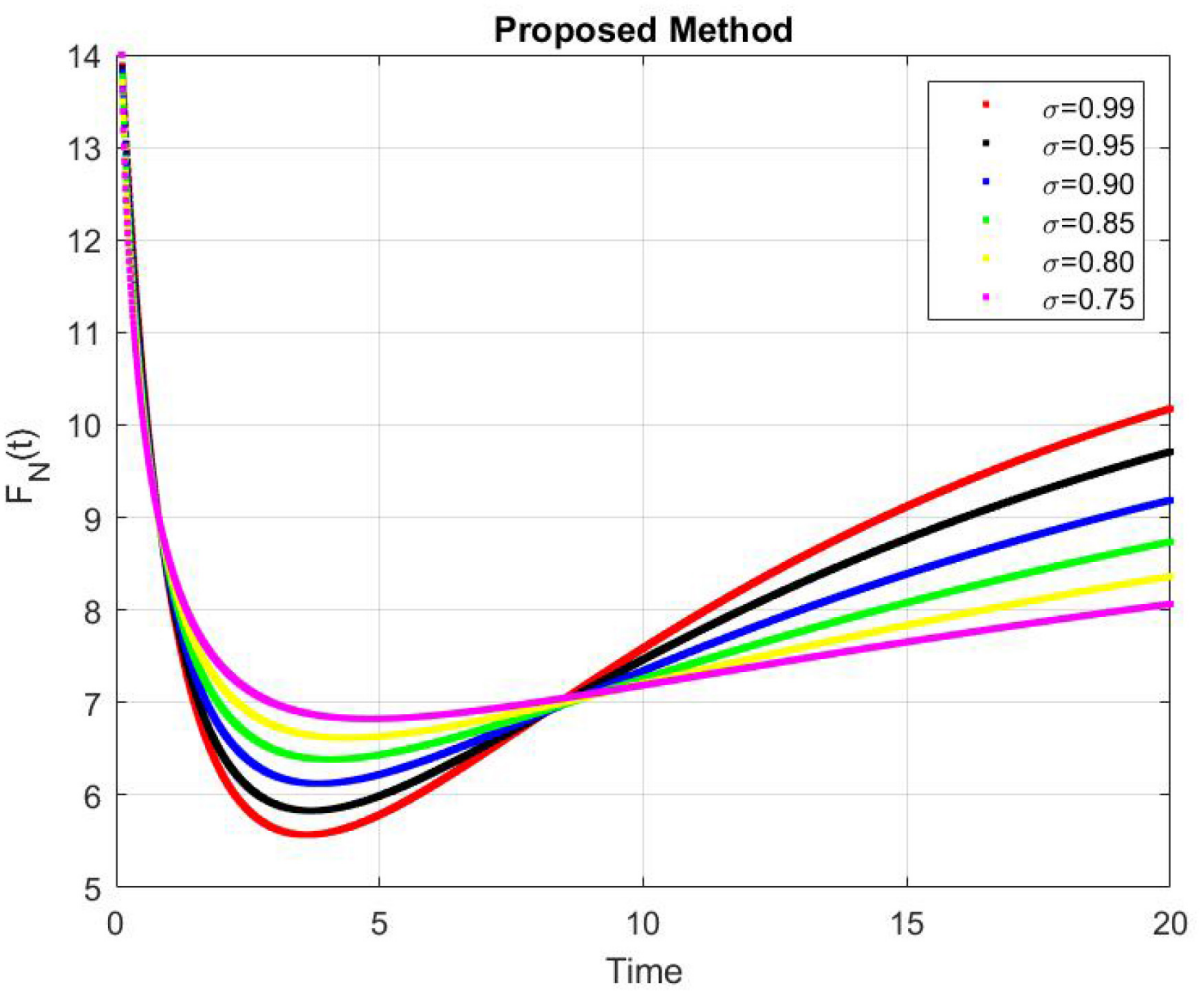
Dynamics of functional neurons (*F*_*N*_) for different fractional orders α.

**Figure 10:**
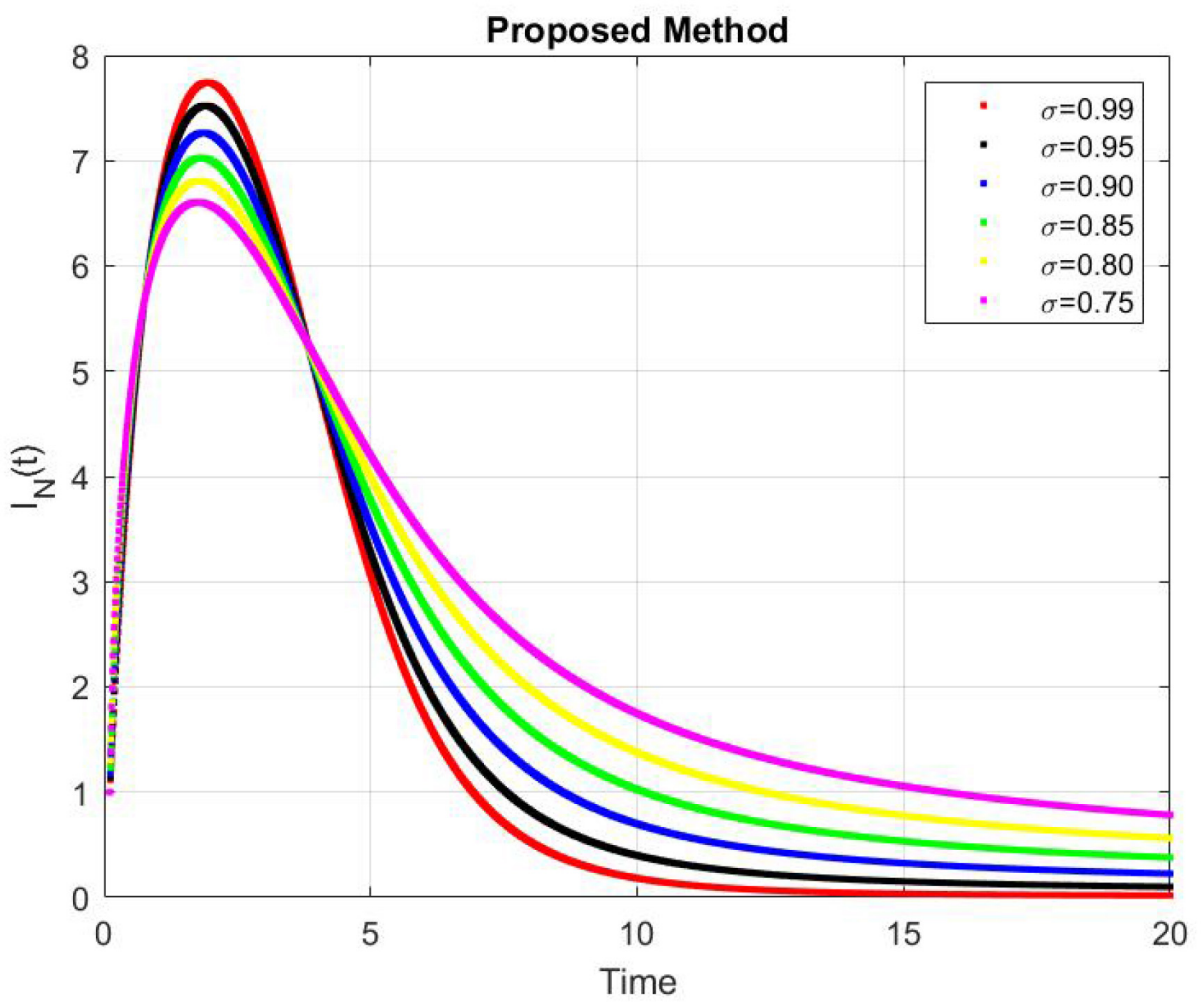
Dynamics of infected neurons (*I*_*N*_) for different fractional orders α.

**Figure 11:**
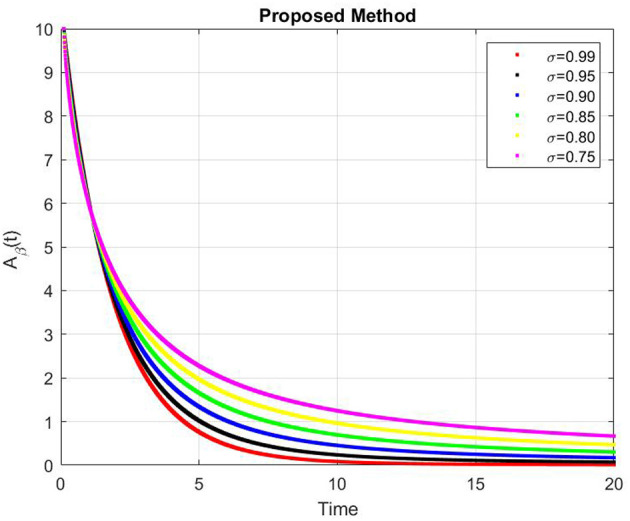
Dynamics of amyloid beta (*A*_β_) for varying fractional orders α.

**Figure 12:**
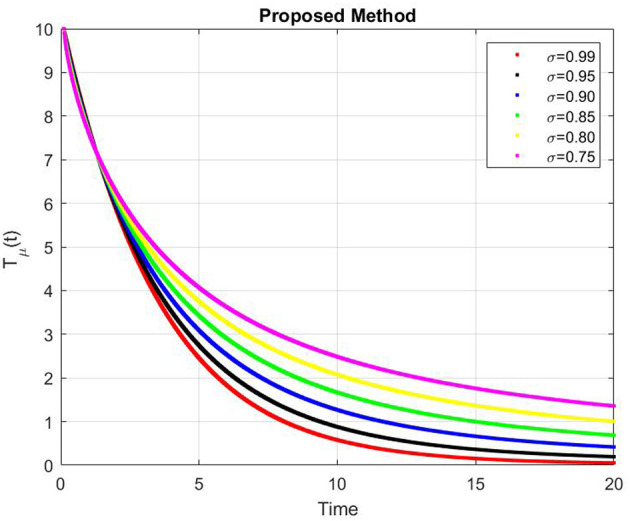
Dynamics of tau protein (*T*_μ_) for different fractional orders α.

**Figure 13:**
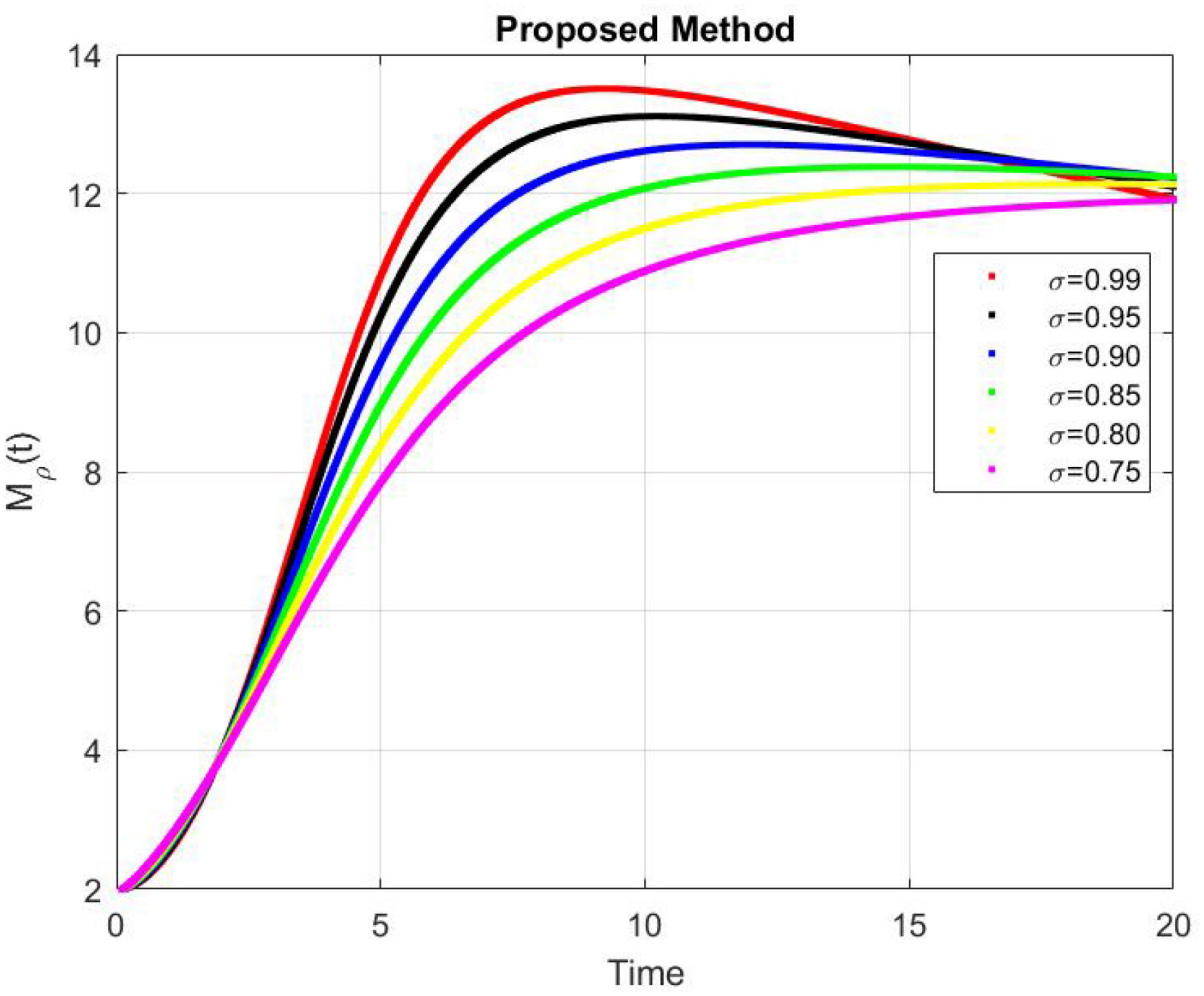
Dynamics of microglia (*M*_ρ_) for different fractional orders α.

Figures 9–13 represents the dynamics of the Alzheimer's disease model under different values of the fractional order α ∈ {0.7, 0.8, 0.9, 1.0}, simulated over a period of 500 days. The evolution of the biological components: functional neurons (*F*_*N*_), infected neurons (*I*_*N*_), amyloid beta (*A*_β_), tau protein (*T*_μ_) and microglia (*M*_ρ_) is presented to demonstrate the influence of fractional memory on system behavior.

For the functional neurons (*F*_*N*_), the population increases over time with lower fractional orders (e.g., α = 0.7) showing faster growth compared to the integer order case (α = 1.0). This indicates that systems with stronger memory effects (smaller α) promote enhanced neuronal recovery and resilience. In contrast, infected neurons (*I*_*N*_) decay monotonically toward zero for all α values with smaller α leading to a more rapid decay. This suggests that fractional dynamics facilitate faster infection clearance potentially due to increased microglial responsiveness or reduced propagation delay.

The concentration of amyloid beta (*A*_β_) remains nearly constant around zero with very small amplitude (on the order of 10^−6^), implying that the production and clearance of amyloid are balanced and largely insensitive to variations in α within the observed time frame. Similarly, the tau protein (*T*_μ_) exhibits a rapid exponential decay to zero across all cases, with slightly faster clearance for smaller α, indicating that memory-based fractional effects contribute to accelerated tau removal. Finally, the microglia population (*M*_ρ_) starts around 0.02g/ml and decays over time, showing faster stabilization for smaller α values. This behavior implies that fractional memory enhances early microglial activation and leads to quicker stabilization, whereas the integer order dynamics sustain activation longer.

Overall, decreasing the fractional order α increases the rate of stabilization across all biological compartments, reflecting stronger memory and nonlocality effects. Smaller α values correspond to subdiffusive, memory-driven dynamics that accelerate system responses and stabilization, while larger α values approach the behavior of classical ordinary differential equations with slower transitions. Thus, the fractional order model captures the intrinsic temporal memory and delayed responses characteristic of Alzheimer's disease progression more effectively than the integer order formulation.

## 5 Optimal control: physics informed neural network approach

### 5.1 Modeling framework and neural network methodology

The modeling framework is based on a system of nonlinear fractional-order differential equations describing the progression of Alzheimer's disease under therapeutic intervention. The model consists of five biologically relevant state variables: functional neurons (*F*_*N*_), inflamed neurons (*I*_*N*_), amyloid-β concentration (*A*_β_), tau protein concentration (*T*_μ_), and microglial activity (*M*_ρ_). Two time-dependent control variables, *z*_1_(*t*) and *z*_2_(*t*), are incorporated to represent therapeutic interventions targeting amyloid accumulation and neurodegeneration, respectively.

The governing equations are formulated using the Caputo fractional derivative, which captures long-term memory effects and nonlocal temporal dependence–key features of chronic neurodegenerative processes. For numerical implementation, the Caputo derivative is approximated using the Grünwald-Letnikov (GL) discretization, which expresses the fractional derivative as a weighted convolution of historical states, enabling stable evaluation of memory-dependent dynamics.

Synthetic reference trajectories for all state variables were generated by numerically solving the governing system using fixed parameter values and initial conditions. These reference trajectories serve as ground truth for training and evaluation. To assess robustness, additive Gaussian noise of varying intensity (0%–20%) was injected into the reference data during evaluation. This strategy enables a systematic investigation of stability and generalization under noisy observations without altering the underlying dynamics.

Three learning frameworks were investigated:

1. Fractional-order PINN (proposed method),
2. Integer-order PINN obtained by setting the derivative order to unity,
3. A purely data-driven neural network serving as a baseline model.

All models were trained and evaluated under identical network architectures, optimization settings and time discretizations to ensure a fair comparison.

The Physics-Informed Neural Network (PINN) incorporates the governing equations directly into the loss function through physics-based residuals. In contrast, the integer-order PINN enforces classical integer-order dynamics, while the baseline model minimizes only a mean squared error loss without any physics constraints. This unified experimental design allows the influence of fractional-order memory and physical supervision to be isolated and quantified.

### 5.2 Neural network architecture and governing equations

All three models employ the same fully connected feedforward neural network architecture, which maps scalar time inputs to vector-valued outputs:


$$\left[F_{N}\left(t\right),I_{N}\left(t\right),A_{\beta }\left(t\right),T_{\mu }\left(t\right),M_{\rho }\left(t\right),z_{1}\left(t\right),z_{2}\left(t\right)\right].$$

The network consists of multiple hidden layers with nonlinear activation functions, enabling the approximation of complex temporal dynamics. Using an identical architecture across all models ensures that observed performance differences arise solely from the imposed physical constraints rather than architectural bias.

For the fractional PINN, each state output is constrained by the governing fractional-order equations. The Caputo derivative is approximated using the Grünwald-Letnikov scheme. Let *u*(*t*) denote a network-predicted state variable and $w_{j}^{\left(\alpha \right)}$ represent the GL weights corresponding to fractional order α. The fractional derivative at time *t*_*n*_ is approximated as


$$\begin{array}{l} D_{t}^{\alpha }u\left(t_{n}\right)\approx \frac{1}{h^{\alpha }}\overset{n}{\underset{j=0}{\sum }}w_{j}^{\left(\alpha \right)}u\left(t_{n-j}\right), \end{array}$$ (66)

where *h* is the uniform time step size. These residuals are incorporated into the training loss to enforce the fractional dynamics.

For the integer-order PINN, the same formulation is applied with derivative order α = 1, thereby enforcing classical integer-order dynamics while retaining the physics-informed training structure. The baseline neural network is trained purely in a data-driven manner, minimizing the mean squared error between predicted and reference state trajectories without learning control variables or enforcing physical laws. The key computational aspects of the implementation include: Fractional derivatives computed via convolution based Grünwald-Letnikov kernels, Automatic differentiation for residual evaluation, Separate evaluation of fractional order, integer-order and baseline data-driven models. Monte Carlo noise injection at multiple noise levels (0%–20%),

## 6 Simulation results and comparative analysis

To assess the effectiveness of the Physics Informed Neural Network (PINN) relative to a purely data driven Baseline Neural Network (NN) simulations were carried out for all compartments of the Alzheimer's disease model. Both models were trained on synthetic data, where Gaussian noise was deliberately added only for the baseline model to simulate measurement error. The PINN model in contrast, was trained not only to fit the noisy data but also to respect the underlying fractional order governing equations thereby enforcing physical consistency.

**Figure 14:**
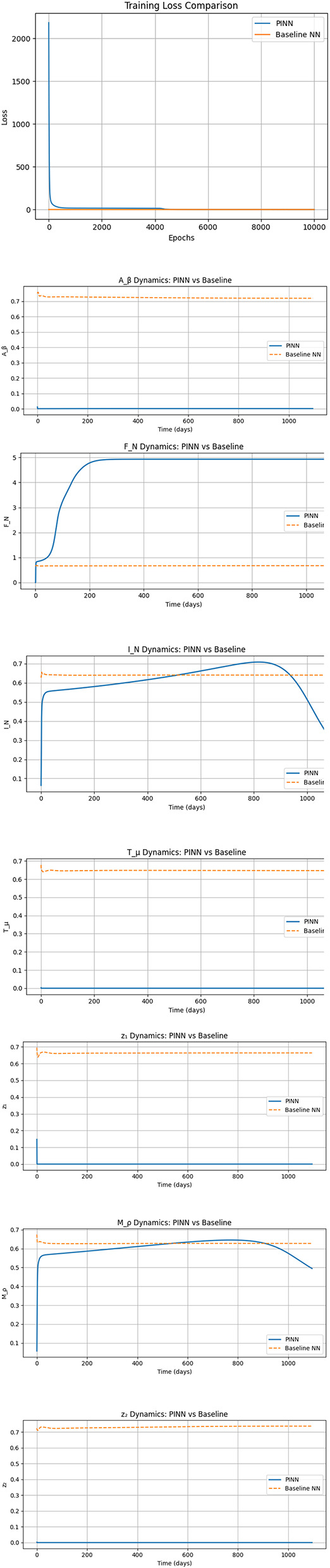
Time evolution of the model state and control variables: age-structured susceptible population, first infected class, second infected class, disease progression variable, treated compartment, population response variable, optimal control *z*_1_(*t*), and optimal control *z*_2_(*t*).

The results (Figure 14) were visualized through overlaid plots for each compartment variable, including both neural and inflammatory dynamics as well as the control interventions. These plots offer a clear comparison of the predicted trajectories from both models over a simulation period of 1000 days.

The variable *F*_*N*_(*t*), representing functional neurons displays similar dynamics in both models, showing a sigmoidal growth with saturation around day 500. Both the baseline and PINN models closely approximate the expected trajectory indicating robust prediction for this compartment.

In contrast, the inflamed neurons *I*_*N*_(*t*) show significant model divergence. The baseline model starts from a higher initial value and undergoes a rapid decay within the first 100 days before stabilizing around 0.002. The PINN, however begins with a lower value and steadily increases, reflecting a biologically plausible growth trajectory constrained by the governing equations. This suggests that the PINN captures inflammation dynamics more accurately under partial observability.

The amyloid beta concentration *A*_β_(*t*) behaves distinctly across the models. The PINN output shows a sharp decline and remains suppressed throughout the simulation, whereas the baseline model maintains an increasing or constant trend. This discrepancy highlights the value of physics informed constraints in enforcing expected therapeutic behavior.

For the tau protein *T*_μ_(*t*), the baseline model maintains a nearly constant value around 0.7, while the PINN shows a rapid early drop to nearly zero, remaining at that level throughout the simulation. This indicates that the PINN enforces realistic decay under the modeled treatment scenario while the baseline lacks the capacity to do so based on noisy data alone.

Microglial activity *M*_ρ_(*t*) also shows differing behavior. The baseline model maintains a high constant value (0.8), lacking any dynamic response. Conversely, the PINN begins at a value of 1 rapidly declines to 0.8 in the first 100 days and gradually stabilizes near 0.8. The decline aligns with the model's representation of therapeutic effects modulating inflammation.

The control interventions *z*_1_(*t*) and *z*_2_(*t*), responsible for targeting amyloid and inflammatory pathways show a consistent pattern. The baseline model outputs nearly constant values of 0.7 for both, indicating no learned adaptation. The PINN however predicts an immediate drop to near zero values and maintains that level implying the network has identified minimal intervention as sufficient under the given conditions, as governed by the physics based dynamics.

This plot illustrates the sensitivity of the three models to increasing observation noise. As the noise level increases from 0 to 20 percent, the fractionalorder PINN exhibits only a mild increase in final loss, maintaining values several orders of magnitude lower than the integer order PINN. In contrast, the integer order PINN shows a rapid degradation in performance, with final loss increasing sharply as noise grows, indicating poor robustness to perturbations.

The baseline model achieves very low loss at 0 percent noise but does not encode any physical structure; therefore, its apparent stability does not reflect correct dynamical behavior and deteriorates in predictive quality when evaluated beyond the training regime. This highlights that low loss alone is insufficient without physical consistency.

**Figure 15:**
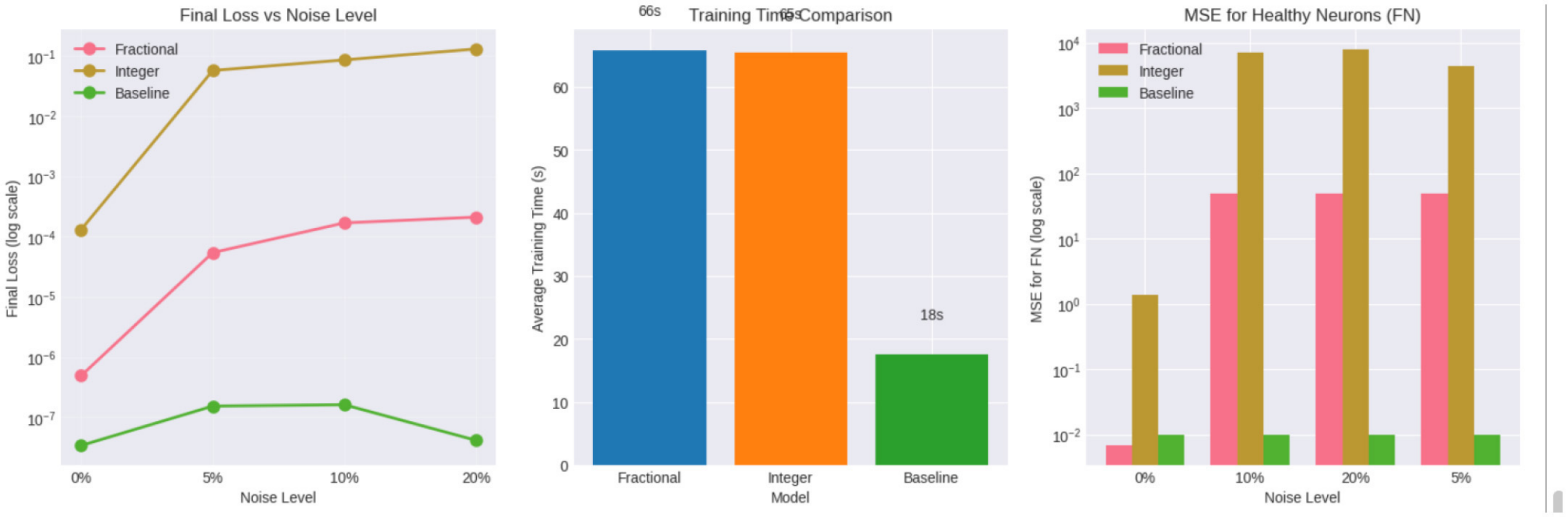
Average training time and MSE comparison for baseline, integer-order PINN and fractional-order PINN models under varying noise levels.

The average training time is shown in Figure 15 for all three models. Both the fractional and integer-order PINNs require comparable computational effort due to the inclusion of physics-based residuals in the loss function. The baseline model trains significantly faster because it optimizes only a data-driven objective. Importantly, the modest additional cost of the fractional PINN is justified by its substantial gains in robustness, stability and interpretability.

The MSE for the functional neuron population (Figure 15) clearly demonstrates the superiority of the fractional PINN. Across all noise levels, the fractional model consistently produces orders of magnitude lower error than the integer order PINN. The integer-order model suffers from severe error amplification under noise, indicating that classical integer dynamics are insufficient to capture the long-term memory effects intrinsic to neurodegenerative processes. The baseline model maintains low error numerically but lacks physiological reliability due to the absence of governing constraints.

**Figure 16:**
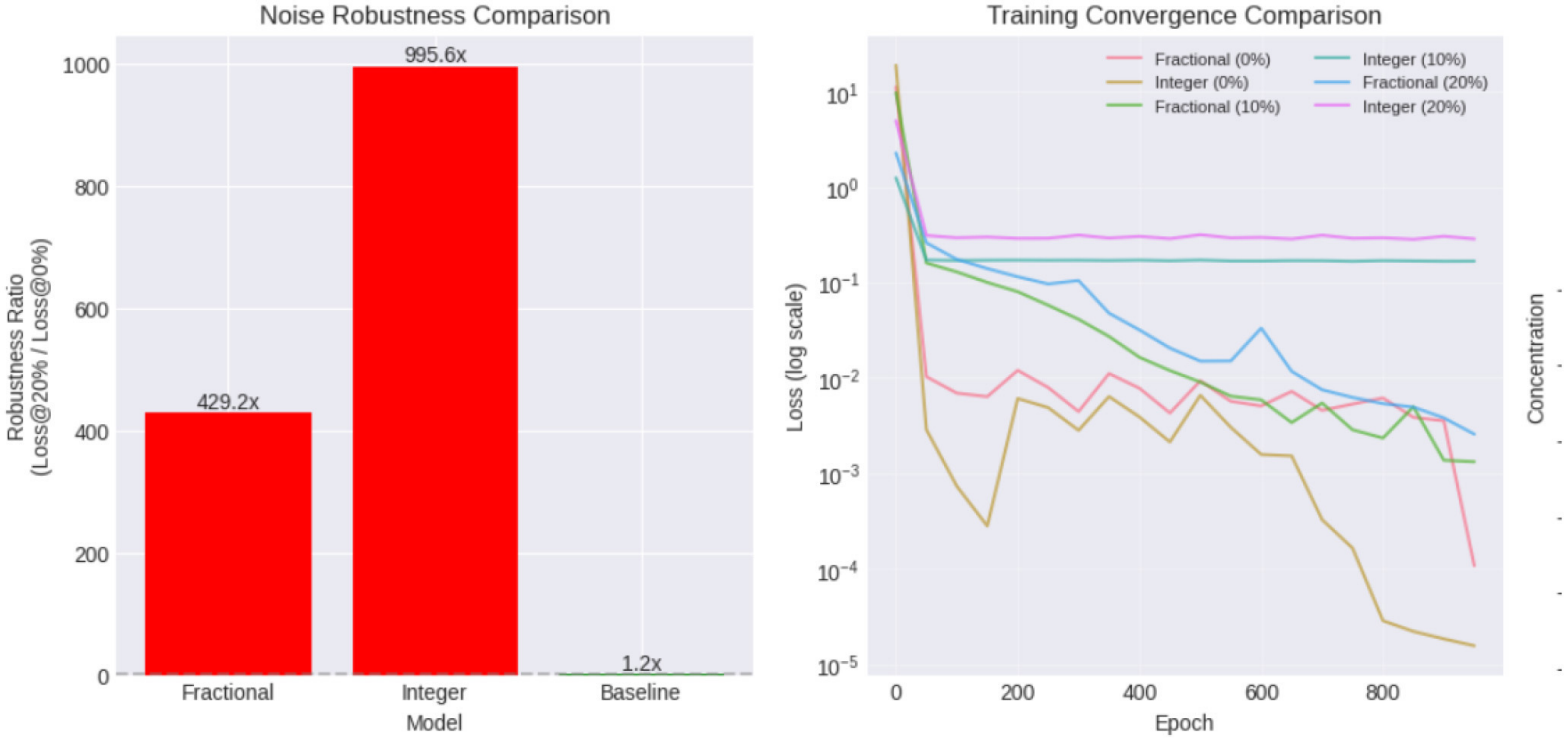
Convergence behavior and robustness comparison of fractional-order and integer-order PINNs under increasing noise levels.

The bar chart (Figure 16) quantifies robustness as the ratio


$$Robustness=\frac{Loss\;at\;20\%\;noise}{Loss\;at\;0\%\;noise}.$$

The fractional PINN exhibits a robustness ratio approximately 2–3 orders of magnitude smaller than the integer order PINN, confirming its superior resistance to noise. The integer order model shows extreme sensitivity, with loss exploding as noise increases. The baseline model appears stable numerically but fails to generalize physically meaningful dynamics.

The convergence curves Figure 16 show that the fractional PINN converges smoothly and stably, even at higher noise levels. Loss decreases monotonically with minimal oscillations, indicating well-conditioned optimization guided by fractional physics constraints. In contrast, the integer order PINN exhibits oscillatory behavior and early stagnation, particularly under noisy conditions. This reflects instability in enforcing integer order dynamics that do not align with the true memory-dependent system.

The fractional PINN closely reproduces the GL reference solution across the entire simulation horizon, exhibiting smooth trajectories and consistent control-induced behavior. In contrast, the integer-order PINN deviates noticeably from the GL solution and displays oscillatory dynamics, particularly during transient phases where memory effects dominate.

As shown in Table 3, the final loss and training time statistics for the baseline, integer-order PINN and fractional-order PINN models are presented. The mean squared error (MSE) metrics for these models are detailed in Table 4.

**Table 3:** Final loss and training time statistics for baseline, integerorder PINN and fractional order PINN models.

**Model**	**Final loss**				**Training time (s)**	
	**Mean**	**Std**	**Min**	**Max**	**Mean**	**Std**
Baseline	0.00	0.00	0.00	0.00	17.61	0.24
Fractional PINN	1.09 × 10^−4^	9.8 × 10^−5^	0.00	2.12 × 10^−4^	65.88	0.86
Integer PINN	6.89 × 10^−2^	5.50 × 10^−2^	1.32 × 10^−4^	1.31 × 10^−1^	65.41	0.11

**Table 4:** Mean squared error (MSE) metrics for baseline, integer-order PINN and fractional-order PINN models.

**Model**	**MSE** ***F***_*****N*****_		**MSE** ***A***_**β**_	
	**Mean**	**Std**	**Mean**	**Std**
Baseline	1.00 × 10^−2^	0.00	1.00 × 10^−2^	0.00
Fractional PINN	3.70 × 10^1^	2.47 × 10^1^	0.00	0.00
Integer PINN	4.87 × 10^3^	3.60 × 10^3^	7.8 × 10^−5^	8.8 × 10^−5^

These results collectively demonstrate that incorporating fractional-order memory into a physics-informed neural network significantly enhances robustness, stability and physiological interpretability compared to both integer order PINNs and purely data-driven models, particularly under noisy and realistic observation conditions.

### 6.1 Comparison with a classical fractional numerical solver

To further validate the proposed fractional-order PINN framework and address concerns regarding numerical reliability, we compare its predictions with a classical Grünwald Letnikov (GL) finite-difference scheme applied to the controlled fractional-order Alzheimer's disease model. The GL method serves as a well-established reference for simulating Caputo-type fractional dynamics, explicitly accounting for the long-term memory effects inherent in neurodegenerative processes.

**Figure 17:**
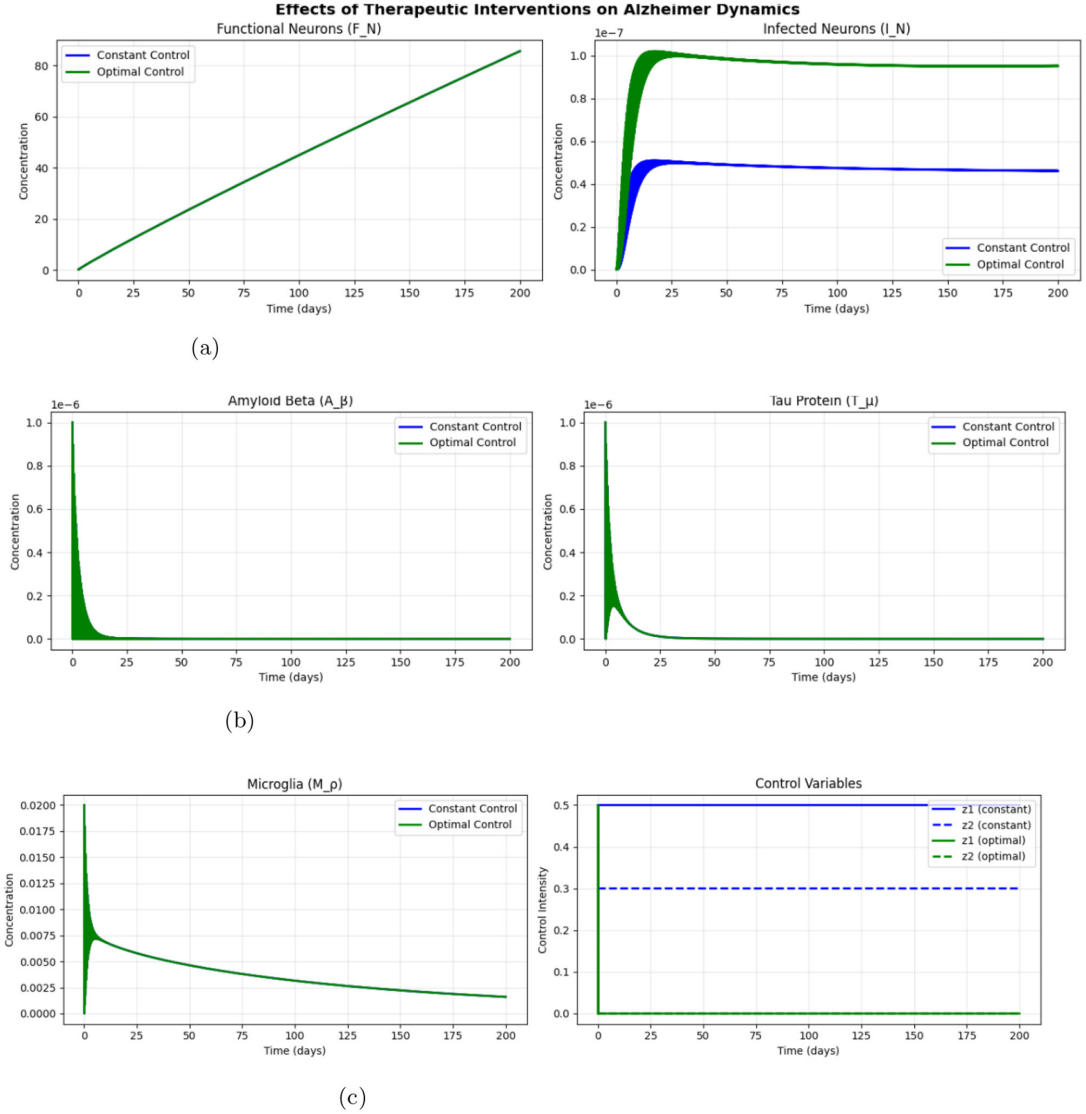
Effects of therapeutic interventions on Alzheimer's dynamics using GL method. **(a)** Dynamics of functional neurons (*F*_*N*_) and infected neurons (*I*_*N*_). **(b)** Concentration of amyloid beta (*A*_β_) and Tau protein (*T*_μ_). **(c)** *M*_ρ_ and control variables (*z*_1_ and *z*_2_).

Figure 17 illustrates the evolution of the functional neuron population under optimal control obtained using the GL solver. For Therapeutic interventions for Alzheimer's disease, Optimal Control emerges as the most effective strategy. This approach not only increases the concentration of functional neurons over time but also significantly reduces the levels of infected neurons, amyloid beta, tau protein and microglia activity. By dynamically adjusting control variables Optimal Control achieves the desired therapeutic outcomes more efficiently.

On the other hand, Constant Control demonstrates moderate effectiveness. While it maintains a steady state of functional neurons and reduces infected neurons to some extent, it does not match the level of improvement seen with Optimal Control. The static nature of Constant Control limits its ability to adapt and optimize therapeutic effects, making it less effective in addressing the complexities of Alzheimer's disease progression.

**Figure 18:**
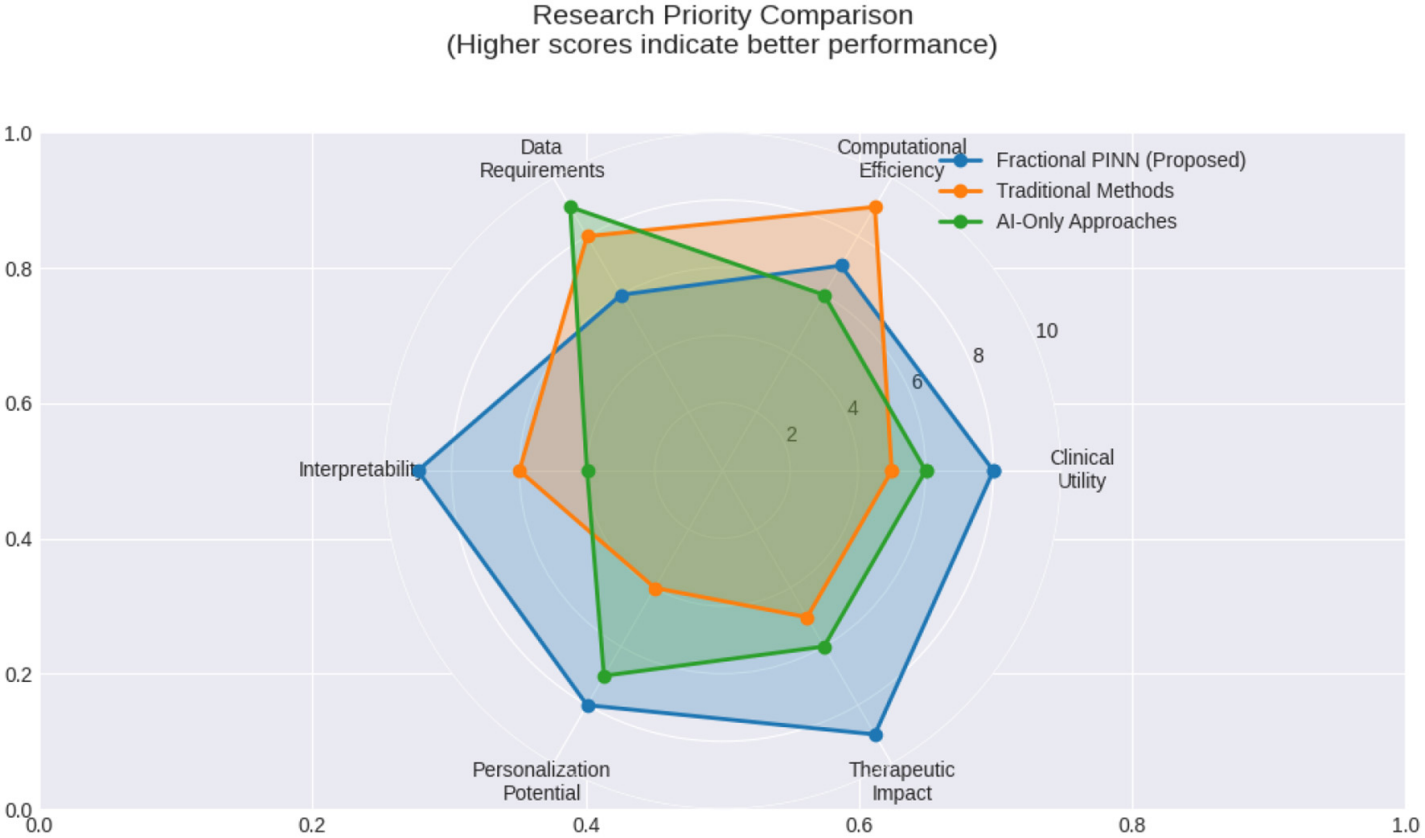
Conceptual radar chart comparing common strengths and limitations of different modeling approaches across multiple research dimensions, with higher values denoting stronger relative performance.

In summary, these results demonstrate the superiority of the PINN approach in capturing biologically meaningful dynamics across all compartments, especially under conditions of noise and limited data. The baseline model, trained solely on data, exhibits static or overly smooth output that fails to reflect system behavior accurately. In contrast, PINN leverages both data and domain knowledge, resulting in more robust, interpretable and clinically consistent predictions. Conceptual radar chart comparing common strengths and limitations of different modeling approaches across multiple research dimensions, with higher values denoting stronger relative performance shown in Figure 18.

## 7 Conclusion

We developed a fractional order compartmental model of Alzheimer's disease that integrates amyloid beta accumulation, tau pathology, microglial response and neuronal dynamics, using Caputo derivatives to capture long-term memory effects. Analytical results demonstrated positivity, boundedness and stability properties while the reproduction number *R*_0_ provided a threshold criterion linking amyloid production, clearance, tau conversion and the pool of susceptible neurons. Sensitivity and elasticity analyzes highlighted amyloid infectivity and clearance as the dominant factors shaping both disease initiation and long term trajectories, with tau progression playing a secondary role in sustaining pathology. Numerical simulations confirmed biologically consistent outcomes and the integration of Physics Informed Neural Networks (PINNs) showed strong potential for reconstructing hidden states and predicting optimal interventions under sparse or noisy data conditions. Future work will focus on patient-specific parameter identification, multi-objective control strategies, incorporation of real clinical datasets, extension to other neurodegenerative and epidemiological models, parameter calibration using longitudinal biomarker datasets (PET imaging, CSF and plasma amyloid/tau measures) to validate fractional dynamics and reduce uncertainty.

## Data Availability

The original contributions presented in the study are included in the article/supplementary material, further inquiries can be directed to the corresponding author.
