# Correction: A Physics Informed Neural Network (PINN) framework for fractional order modeling of Alzheimer's disease

**DOI:** 10.3389/fninf.2026.1821637

**Published:** 2026-03-19

**Authors:** Adnan Mehmood, Muhammad Farman, Farkhanda Afzal, Kottakkaran Sooppy Nisar, Mohammed Altaf Ahmed, Mohamed Hafez

**Affiliations:** 1 CEME, National University of Sciences and Technology (NUST), Islamabad, Pakistan; 2 Department of Mathematics, Mathematics Research Center, Near East University, Northern Cyprus, Türkiye; 3 Research Center of Applied Mathematics, Khazar University, Baku, Azerbaijan; 4 Military College of Signals (MCS), National University of Sciences and Technology (NUST), Islamabad, Pakistan; 5 Department of Mathematics, College of Science and Humanities, Prince Sattam bin Abdulaziz University, Al Kharj, Saudi Arabia; 6 Department of Computer Engineering, College of Computer Engineering & Sciences, Prince Sattam Bin Abdulaziz University, Al Kharj, Saudi Arabia; 7 Faculty of Engineering and Quantity Surviving, INTI International University Colleges, Nilai, Malaysia; 8 Faculty of Management, Shinawatra, Pathum Thani, Thailand

**Keywords:** Alzheimer's disease, health system, machine learning, optimal control, Physics Informed Neural Networks

In the published article, in the Funding and Acknowledgments statements, an incorrect grant number was provided for Prince Sattam bin Abdulaziz University. The correct number should have been “(PSAU/2025/01/34222).” The corrected Funding and Acknowledgments statements appear below.

**Funding**

The author(s) declared that financial support was received for this work and/or its publication. The authors extend their appreciation to Prince Sattam bin Abdulaziz University for funding this research work through the project number (PSAU/2025/01/34222).

**Acknowledgments**

The authors extend their appreciation to Prince Sattam bin Abdulaziz University for funding this research work through the project number (PSAU/2025/01/34222).

In the published article, the Present Address superscript symbol and details were erroneously omitted for author FA. The Present Address should have been included as: “Farkhanda Afzal, School of Electrical Engineering and Computer Science, National University of Sciences and Technology, Islamabad, Pakistan”.

The original version of this article has been updated.

